# Investigating the temporal dynamics and modeling of mid-level feature representations in humans

**DOI:** 10.1162/IMAG.a.1207

**Published:** 2026-04-27

**Authors:** Agnessa Karapetian, Alexander Lenders, Vanshika Bawa, Martin Pflaum, Raphael Leuner, Gemma Roig, Kshitij Dwivedi, Radoslaw M. Cichy

**Affiliations:** Department of Education and Psychology, Freie Universität Berlin, Berlin, Germany; Charité – Universitätsmedizin Berlin, Einstein Center for Neurosciences Berlin, Berlin, Germany; Bernstein Centre for Computational Neuroscience Berlin, Berlin, Germany; Faculty of Biology, Albert-Ludwigs-Universität Freiburg, Freiburg, Germany; Fraunhofer Institute for Laser Technology ILT, Aachen, Germany; Department of Mathematics and Computer Science, Freie Universität Berlin, Berlin, Germany; Department of Computer Science, Goethe Universität Frankfurt, Frankfurt am Main, Germany; Berlin School of Mind and Brain, Faculty of Philosophy, Humboldt-Universität zu Berlin, Berlin, Germany

**Keywords:** mid-level features, visual perception, scene processing, encoding, EEG, CNN

## Abstract

Visual perception unfolds through a hierarchy of transformations, beginning with the extraction of low-level features, such as edges, and culminating in the representation of high-level features such as object categories. While the processing of low- and high-level features is well studied, the intermediate transformations, that is, mid-level features, remain poorly understood. Here, we introduce a stimulus set of naturalistic 3D-rendered images and videos with ground-truth annotations for five candidate mid-level features (reflectance, scene depth, world normals, lighting, and skeleton position) alongside for one low-level feature (edges) and for one high-level feature (action identity). To determine when these features are processed in the brain, we collected electroencephalography (EEG) responses during stimulus presentation and trained linearized encoding models to predict EEG responses from the annotations. We first showed that candidate mid-level features were best represented between ~100 and 250 ms post-stimulus, between low- and high-level features, and consistent with a bridging role linking sensory and semantic processing. We then assessed convolutional neural networks (CNNs) as models of mid-level feature processing in humans and observed that although their hierarchies were shallower, they exhibited a comparable processing order for mid-level but not low- or high-level features, only for videos. Together, our results support the view that mid-level features are tied to surface- and shape-related processing and establish 3D-rendered stimuli with annotations as a valuable tool for investigating mid-level vision in biological and artificial neural networks.

## 1 Introduction

Theories of vision commonly characterize object and scene perception as hierarchical processes, transforming retinal input into progressively abstract and complex representations that support visual recognition, categorization, and decision making (Biederman, 1987; DiCarlo et al., 2012; Henderson & Hollingworth, 1999; Marr, 1982; Riesenhuber & Poggio, 1999; Yamins & DiCarlo, 2016). This hierarchy is often described in terms of the processing of low-, mid-, and high-level features. Low-level features, such as edges and contrast, emerge within ~50–100 ms in early visual cortex, primarily within V1 (Cichy et al., 2014; Di Russo et al., 2002; Hubel & Wiesel, 1959). High-level features encode semantics such as object, action, or scene identity and are associated with higher-order regions, including inferotemporal cortex (IT) for objects (DiCarlo et al., 2012) or parahippocampal place area (PPA) and retrosplenial cortex (RSC) for scenes (Epstein & Baker, 2019). Their latencies vary with task demands and stimulus properties (Contini et al., 2017), ranging from ~100 ms for coarse discrimination and recognition (Di Russo et al., 2002; Vogel & Luck, 2000) to ~200–450 ms for action recognition (Isik et al., 2018).

In contrast, the intermediate transformations that give rise to mid-level feature representations remain less well understood (Anderson, 2020; Peirce, 2015). We define mid-level features operationally as stimulus properties that are neither fully captured in early visual areas (e.g., V1 within ~100 ms) nor uniquely represented in higher-order semantic visual regions. This broad definition allows for a data-driven exploration of candidate features without prematurely committing to a single theoretical framework (Gelfert, 2016). Guided by classical theories of vision (Biederman, 1987; Marr, 1982; Riesenhuber & Poggio, 1999), studies of traditional mid-level features (e.g., textures, contours, and 3D surfaces) have linked their representations to areas V2, V3, and V4, and, for certain scene-related surface properties, to PPA and RSC (Choo & Walther, 2016; Dumoulin & Hess, 2007; Jagadeesh & Gardner, 2022; Lescroart & Gallant, 2019; Long et al., 2018; Pasupathy & Connor, 1999, 2001; Walther et al., 2011). These findings provide a strong foundation regarding the cortical loci of mid-level features, yet the temporal dimension remains largely unexplored.

Moreover, progress in the understanding of mid-level feature processing is limited by four methodological constraints. First, most research on mid-level features has been performed using object stimuli, raising concerns about generalizability to naturalistic inputs with multiple objects, complex spatial layouts, and rich statistical dependencies (Epstein & Baker, 2019; Groen et al., 2017). Second, ground-truth annotations of mid-level features are typically available only for artificial stimuli, so studies investigating naturalistic inputs often rely on model approximations (Zamir et al., 2018) or data-driven feature representations (e.g., deep neural network layer activations). These approximations are prone to error, are noisy, and may fail to accurately represent human perception, particularly for mid-level features (Geirhos et al., 2022). Third, most stimulus sets focus on only one or two mid-level features, often shape or texture (e.g., Backus et al., 2001; Freeman et al., 2013), overlooking the diversity of mid-level features and their natural co-occurrences, potentially missing how the visual system exploits inherent statistical regularities in stimuli (Scholte & Haan, 2025). Fourth, most studies rely on static images, leaving open how motion affects mid-level processing, despite evidence that mid-level areas such as V3, V4, and V5/MT simultaneously process motion and shape, and that motion impairments can disrupt shape perception (Scholte & Haan, 2025).

To address these four constraints, we created a novel stimulus set of 3D-rendered naturalistic scenes as still images and dynamic videos with accompanying ground-truth annotations (Epic Games, 2019). This stimulus set features characters performing various actions, addressing scene, object, and action perception jointly; provides stimuli along with noise- and error-free ground-truth annotations for mid-level features; covers multiple candidate mid-level features—reflectance, lighting, world normals, scene depth, and skeleton position—, as well as a low-level feature, edges, and a high-level feature, action identity, and it does so for both static and dynamic stimuli.

Thus equipped, we first assessed mid-level feature processing in the human brain by characterizing its temporal dynamics in humans via electroencephalography (EEG) and linearized encoding models (Lescroart & Gallant, 2019; Naselaris et al., 2011). To evaluate motion effects, we collected EEG responses to both static images and dynamic videos and evaluated how the encoding results differed across these conditions. We then explored whether convolutional neural networks (CNNs), commonly used as models of the visual system (Cichy & Kaiser, 2019; Doerig et al., 2023), represent mid-level features in ways comparable with those observed in the human brain. CNNs are promising computational models since they learn representations from large-scale, naturalistic datasets, and reliably predict neural activity across the visual cortex, including mid-level areas such as V4 (Yamins & DiCarlo, 2016). However, despite their predictive power, it remains unclear to what extent CNN representations encode mid-level features in a similar way to the brain and reflect its processing hierarchy.

Taken together, our work pursues three goals: (1) characterize the temporal dynamics of mid-level vision under naturalistic conditions, (2) determine how motion affects mid-level feature processing, and (3) assess whether mid-level feature processing is hierarchically aligned between humans and CNNs, a common computational model of the visual system.

## 2 Materials and Methods

### 2.1 Participants

The study consisted of two experiments: an image experiment, where one set of participants viewed static images of scenes, and a video experiment, where another set of participants viewed dynamic videos of the same scenes. Fifteen healthy participants took part in the image experiment (mean age: 23.5 years, *SD* = 2.58; 9 female, 6 male), and 20 healthy participants took part in the video experiment (mean age: 25.15 years, *SD* = 4.33; 17 female, 3 male). All participants had normal or corrected-to-normal vision. All participants provided their informed consent after getting acquainted with the study protocol. The study was approved by the ethics committee of Freie Universität Berlin.

### 2.2 Stimuli and ground-truth annotations

**Fig. 1.**
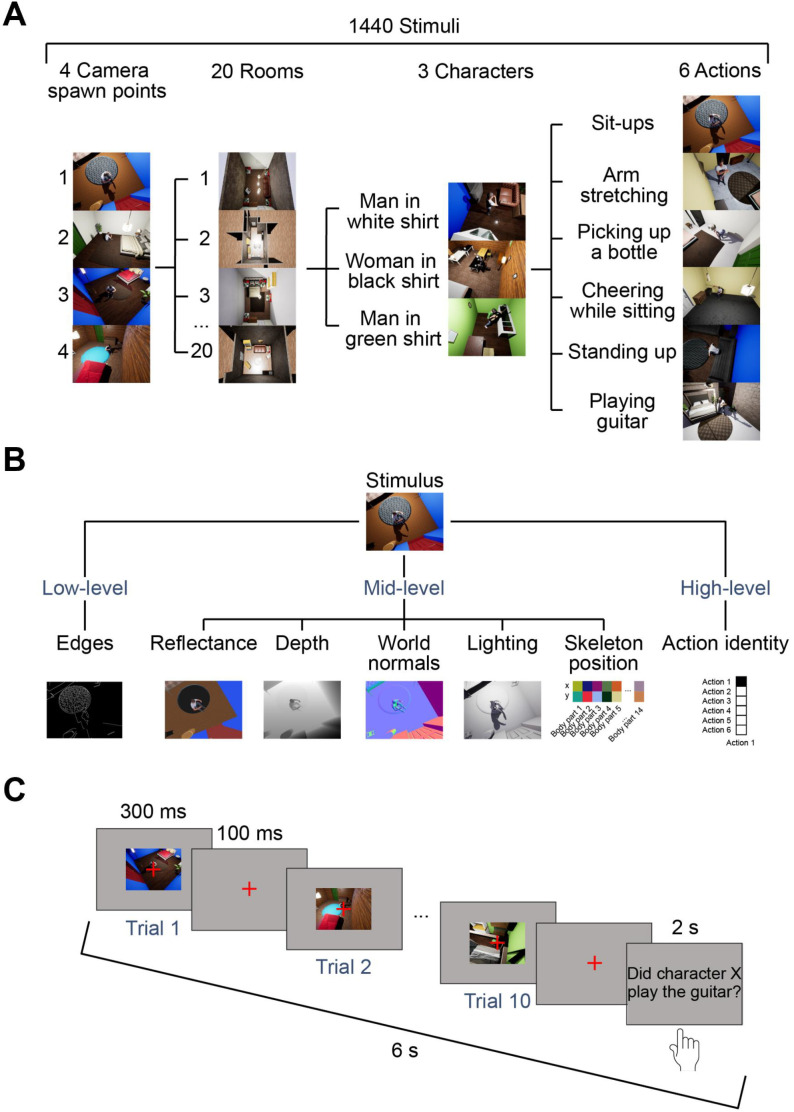
Stimuli, ground-truth annotations, and paradigm. (A) Examples of stimuli created in a game engine (Epic Games, 2019), available as images and 300-ms videos. The stimulus set contained 1440 samples, created by using 4 camera spawn points, 20 rooms, 3 characters, and 6 actions. (B) Example annotations created in the game engine for the low-, mid-, and high-level features of interest. (C) Paradigm used in the experiment. Each sequence lasted 6 seconds, consisting of 10 trials of 400 ms each (300 ms long stimulus presentation followed by 100 ms of intertrial interval (ITI)) and 2 seconds of response time. The participants’ task was to indicate whether a specific character (either man in white shirt, woman in black shirt, or man in green shirt) was shown playing the guitar at least once in the sequence.

We created the stimuli (Fig. 1A) and ground-truth annotations (Fig. 1B) using Unreal Engine (Epic Games, 2019; see Supplementary Note S1 for details of the rendering pipeline). Each stimulus depicted a naturalistic scene where a character performed an action inside a room. The stimulus set consisted of scenes showing 1 of 3 different characters (a woman in a white T-shirt, a man in a black T-shirt, and a man in a green T-shirt), performing 1 of 6 different actions (arm stretching, cheering while sitting, picking up a bottle from the floor, sit-ups, standing up, and playing guitar), in 1 of 20 different rooms (each having a different layout and objects) and filmed from 1 of 4 different camera spawn points (i.e., locations where the camera is filming from approximately), resulting in 1440 different stimuli. The stimuli were available as 520 × 390 pixel still images and 300-ms videos of the same dimensions with a frame rate of 30 fps (i.e., containing 9 frames).

For every stimulus and frame (for videos), we obtained ground-truth annotations of 5 candidate mid-level features: reflectance, lighting, world normals, scene depth, and skeleton position (Fig. 1B; see Supplementary Table S1 for detailed descriptions of annotations). The mid-level features were selected based on classical theories of visual perception linking mid-level processing to surfaces, depth, and contours (Biederman, 1987; Marr, 1982; Riesenhuber & Poggio, 1999) and on their availability for being rendered from the game engine. In addition, we included annotations for two reference features to contextualize the mid-level features: one low-level feature (edges) and one high-level feature (action identity). We refer to these annotations as “ground truth” only in the sense that they are exact, noise-free outputs from the game engine. This does not imply that they constitute ground truth for how biological vision represents information.

We rendered the ground-truth annotations for four of the features (reflectance, lighting, world normals, and scene depth). The rendered annotations for the colorful features (reflectance and world normals) were of size 520 × 390 × 3, representing pixel-wise, RGB channel-wise ground-truth information for the given feature. The annotations for the grayscale features (lighting and scene depth) were of size 520 × 390.

For the remaining three features (edges, skeleton position, and action identity), we obtained the annotations differently. To obtain edge annotations, we used the Canny algorithm (Canny, 1986) as implemented in OpenCV (Bradski, 2000). This resulted in a matrix of size 520 × 390, representing pixel-wise edge information for each stimulus and frame (for videos). For skeleton position, that is, the x and y coordinates of the character’s 14 body parts (head, neck, left upper arm, right upper arm, left lower arm, right upper arm, left hand, right hand, left thigh, right thigh, left calf, right calf, left foot, right foot), we obtained the information from the meta-data files provided by the engine, resulting in a matrix of size 14 × 2. Lastly, for action identity, that is, the action performed by the character, we created one-hot vectors for each of the six actions and used them to encode the action information for each of the scenes. This resulted in one ground-truth annotation per stimulus and feature for images, and one ground-truth annotation per stimulus, feature, and frame for videos.

Finally, for training and evaluating encoding models, the stimuli and their annotations were split into training, test, and validation sets, each respectively containing 1080, 180, and 180 samples. The training set included scenes spanning 15 rooms, 6 actions, 4 camera angles, and 3 characters. The test and validation sets each included scenes from 5 rooms not shown in the training set, 6 actions, 2 camera angles (the same as in the training set: two for the test set and the two others for the validation set), and 3 characters.

### 2.3 Paradigm and experimental design

In both image and video experiments, participants completed a rapid serial visual presentation (RSVP) task (Fig. 1C). Each of the image and video experiments consisted of 22 self-initiated runs assigned to training, test, and validation sets (10, 10, and 2 runs, respectively). The training and test runs alternated every run, and the validation runs were added in between (one in the middle and one in the end). The training and test runs contained 54 sequences of 10 trials each, while the validation set contained 45 sequences of 10 trials. In total there were 5400 trials in the training set (5 trial repetitions for each of the 1080 stimuli), 5400 trials in the test set (30 trial repetitions for each of the 180 stimuli), and 900 trials in the validation set (5 trial repetitions for each of the 180 stimuli). The order of the presented stimuli was randomized for every run separately. Each training and test run lasted approximately 5.5 minutes, and each validation run lasted approximately 4.5 minutes, resulting in a total session duration of about 2 hours.

On each trial, participants viewed a stimulus for 300 ms (a still image in the image experiment or a 300-ms video in the video experiment) at a visual angle of 15.8˚ by 10.6˚, which was followed by an intertrial interval (ITI) of 100 ms showing a gray screen, totaling 4 seconds of presentation time for each sequence of 10 trials. We selected 300 ms as trial duration because it is short enough to minimize the effect of eye movements on EEG data, but long enough for the movement information to still be present in the videos. A red fixation cross was at the center of the screen throughout the stimulus presentation and the ITI to encourage participants to fixate. After these 4 seconds of presentation time and prior to the start of the next sequence, participants had 2 seconds to indicate whether they saw the target character (a woman in a black T-shirt, a man in a white T-shirt, or a man in a green T-shirt) play the guitar by pressing “J” on odd runs and “F” on even runs if they did, and the opposite key if they did not. The target action remained the same throughout the experiment, while the target character varied across runs. For training, test, and validation runs, the target characters were defined in the same repeating order: the man in the white T-shirt, the man in the green T-shirt, and the woman in the black T-shirt. Participants were also invited to blink during the response period.

Prior to data collection, in order to familiarize themselves with the task, participants completed 7 practice runs each containing three sequences.

The experiment was programmed and performed using PsychToolbox (Brainard, 1997) in MATLAB (2021a).

### 2.4 EEG recording and preprocessing

During the image and video experiments, we recorded electroencephalography (EEG) data using a 64-channel actiCAP active electrode system from BrainVision with a sampling rate of 1000 Hz. The signal was amplified using the BrainAmp amplifier. The electrodes on the Easycap 64-electrode system were arranged based on the 10–10 system. The participants wore actiCAP elastic caps, connected to 64 active scalp electrodes, plus 1 ground (AFz) and 1 reference electrode (FCz). The signal was filtered online between 0.03 and 100 Hz to eliminate baseline drift and high-frequency noise.

Offline, we preprocessed the raw EEG voltage signals using the MNE package (version 1.2.2; Gramfort et al., 2013). Preprocessing included filtering line noise with a notch filter (50 Hz), independent component analysis (ICA) to remove artifacts such as eye movements and muscle activity, low-pass filtering (25 Hz), resampling to 50 Hz (70 time points), selecting 19 posterior (parietal and occipital) channels (i.e., channels overlaying the visual cortex) for computational efficiency, and dividing the sequences into epochs surrounding individual stimuli. This resulted in epochs starting from 400 ms before stimulus onset to 980 ms after stimulus onset, which we baseline corrected based on a 100 ms pre-stimulus window.

To control for differences in noise levels across channels, we applied multivariate noise normalization (MVNN; Guggenmos et al., 2018). In the first step, we estimated the noise covariance matrix using the Ledoit–Wolf shrinkage estimator. This matrix quantifies the trial-to-trial noise variance of each EEG channel and the noise covariances between channel pairs, providing a regularized estimate of shared noise structure across channels.

We computed the noise covariance matrix from repeated trials of the same stimulus in the training set, separately for each stimulus and at each time point. This procedure assumes that stimulus-evoked activity is approximately constant across repetitions, such that trial-to-trial variability predominantly reflects noise rather than signal. Averaging across time points and stimuli yielded a single noise covariance matrix. In the second step, we applied the inverse square root of this matrix as a whitening transformation, multiplying the trial-specific preprocessed EEG responses by this matrix. This linear transformation whitens the estimated noise covariance, resulting in decorrelated and unit-variance noise across channels. The noise covariance matrix estimated on the training set was applied to the training, validation, and test sets.

After preprocessing, each trial contained, at every time point, a pattern of 19 channel activations, hereafter referred to as EEG responses, which were used in all subsequent EEG analyses.

### 2.5 Decoding

To determine and compare the signal-to-noise ratios of our image and video EEG responses, we assessed the decodability of individual scenes by performing pairwise decoding (Cichy et al., 2014; Grootswagers et al., 2017) on subject-level preprocessed EEG data. For this, we used a linear support vector machine (SVM; Vapnik, 1995) in scikit-learn (Pedregosa et al., 2011).

We performed pairwise decoding on every pair of scenes from the test set of the encoding analysis, independently for image and video EEG responses, and for every subject and every time point. We performed the analysis on the 180 scenes from the encoding test set given that it had the largest number of trial repetitions per scene (30).

This procedure involved two steps. First, to increase the signal-to-noise ratio for each scene, we created 6 pseudotrials from the 30 trial repetitions by averaging over randomly formed bins of 5 trial repetitions. This resulted in 6 pseudotrials per scene and, therefore, 12 pseudotrials for each pairwise combination. Second, for each pairwise combination, we employed a stratified sixfold cross-validation procedure (Cichy et al., 2014; Grootswagers et al., 2017). Using this procedure we first randomly split the 12 pseudotrials from each pairwise combination into a decoding training set and a decoding test set. We then trained the SVM on the decoding training set to classify every pair of scenes and evaluated it on the decoding test set. We obtained a symmetric matrix of pairwise decoding accuracies for all 180 × 180 pairs of scenes, of which we took the lower diagonal, flattened it, and averaged across all entries, resulting in 1 average decoding accuracy per fold. We repeated this analysis for every fold (i.e., 6 times), using a different decoding training and test set each time, and averaged over the decoding accuracies across folds.

After performing this analysis on every time point, for each participant, and for both images and videos, we averaged the decoding accuracies across participants and obtained one decoding time course for images and one for videos. We compared these two time courses by subtracting the video time course from the image time course, providing us with a measure of difference between the decodability and, therefore, of signal-to-noise ratio, of our two datasets.

### 2.6 Encoding

To determine when the candidate low-, mid-, and high-level features are processed in the brain and whether there is a comparable processing hierarchy across the layers of CNNs trained on vision tasks, we trained and evaluated linearized encoding models (Naselaris et al., 2011). We give an overview of the workflow before detailing each step. First, we preprocessed our ground-truth annotations into a suitable format for training the encoding models. Then, for CNNs, we extracted and preprocessed activations at different layers throughout the networks. On this basis, separately for EEG and CNNs and for images and videos, we used ridge regression (Hastie et al., 2009) as the encoding model to predict the EEG responses at every time point, or CNN activations at every layer, from our low-, mid-, and high-level feature ground-truth annotations. We then assessed the accuracy of each encoding model by correlating the predicted and true EEG responses or CNN activations, which revealed when or at which layer low-, mid-, and high-level features are represented in the human brain and CNNs. Lastly, we collected the encoding peak latencies for EEG and peak layers for CNNs for all features and correlated them, revealing similarities and dissimilarities in the processing hierarchy of low-, mid-, and high-level features.

#### 2.6.1 Preparing the ground-truth annotations

**Fig. 2.**
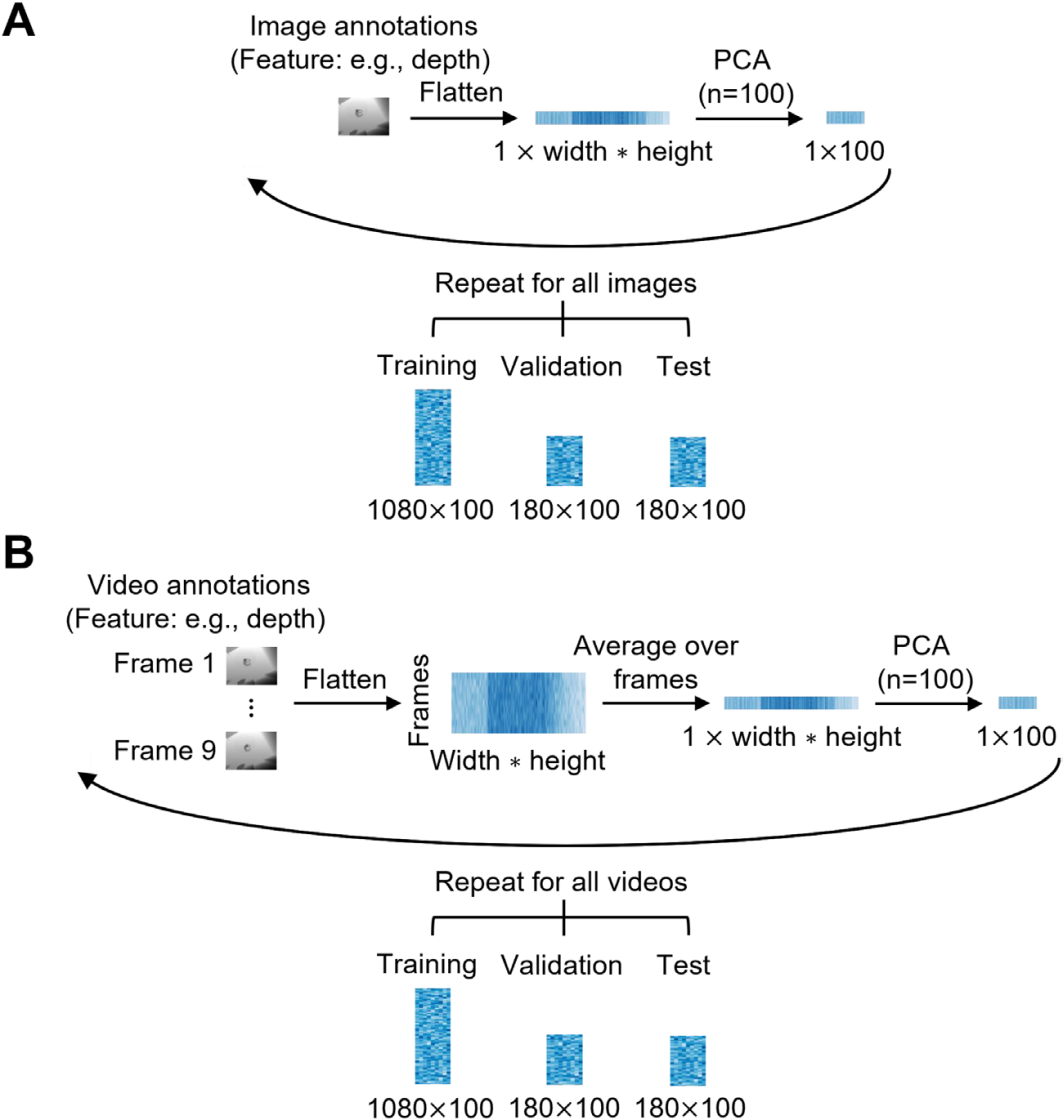
Preparation of ground-truth annotations. (A) Image annotations. For each feature separately, the ground-truth annotation for every image was (1) flattened across the width, height, and, if applicable, RGB dimensions, and (2) reduced in dimensionality via PCA (100 components) for the higher-dimensional features. Repeating the procedure over all images resulted in vectors of size 100 for every image from the training set (1080 samples), validation set (180 samples), and test set (180 samples). (B) Video annotations. For each feature separately, the ground-truth annotations for all 9 frames were (1) flattened across the width, height and if applicable, RGB dimensions, (2) averaged over the 9 frames, and (3) reduced in dimensionality via PCA (100 components) for the higher-dimensional features. Repeating the procedure for all videos resulted in vectors of size 100 for every video from the training set (1080 samples), validation set (180 samples), and test set (180 samples).

To put the image ground-truth annotations of the low-, mid-, and high-level features into a format suitable for the encoding analysis, we prepared them by applying two preprocessing steps (Fig. 2A). First, for each feature, except skeleton position and action identity, and each stimulus in the training, validation, and test sets, we flattened the annotations along the width, height, and for colorful features, RGB dimensions. For skeleton position, we flattened the annotations across x/y coordinates to transform them into 1D vectors. Action identity was already in a 1D format. Second, to improve the computational efficiency of the encoding analysis, we reduced the dimensionality of the annotations for the higher-dimensional features, that is, edges, reflectance, lighting, world normals, and scene depth. For this, we employed a principal component analysis (PCA) with a linear kernel and 100 components. We fitted PCA on the training set activations and projected the training, validation, and test sets onto the resulting components. PCA was not employed on the 2 lower-dimensional features, that is, skeleton position and action identity, as they already contained less than 100 components (28 for skeleton position and 6 for action identity).

To prepare the video ground-truth annotations (Fig. 2B), we applied three preprocessing steps. First, we flattened the annotations across the width, height, and when applicable, RGB dimensions for each feature (except for skeleton position and action identity), stimulus and video frame, and across x/y coordinates for skeleton position. Second, for each feature and stimulus, we averaged over the frames. Third, for each stimulus, we applied PCA with 100 components on the averaged annotations for the 5 higher-dimensional features.

#### 2.6.2 Extracting and preparing the CNN activations

To train and evaluate encoding models predicting CNN activations from the ground-truth annotations, we first extracted the hidden layer activations and then preprocessed them for the encoding analysis.

The details of the first step were as follows. We first extracted hidden layer activations from two CNNs: one trained on scene classification using images as inputs, referred to as image CNN, and one trained on action classification using videos as inputs, referred to as video CNN.

For the image CNN, we used an instance of ResNet-18 (He et al., 2015) pre-trained on Places365-Standard (Zhou et al., 2018), a dataset of more than 1.8 million scene images across 365 scene categories. We extracted activations from its hidden layers in response to all the images from our stimulus set. Before extracting the activations, we preprocessed the images to put them in a suitable format for the network. First, we resized the smaller side to 256 pixels and applied a center crop to obtain 224 × 224 pixels. We then normalized pixel values by subtracting the ImageNet mean and dividing by the ImageNet standard deviation for each channel, ensuring consistency with the statistics of the pretrained network. Afterwards, we fed the preprocessed images to the network and extracted the activations from the last layers of eight residual blocks (referred to as layer 1.0, layer 1.1, layer 2.0, layer 2.1, layer 3.0, layer 3.1, layer 4.0, and layer 4.1) using Torch FX in PyTorch, yielding activations from eight layers throughout the network hierarchy for the training, validation, and test sets of images.

For the video CNN, we used an instance of 3D ResNet-18, a network that employs 3D convolutions on the spatiotemporal video volume (Tran et al., 2018). It was pre-trained on the Kinetics400 dataset comprising up to 650,000 videos that cover 400 human actions (Kay et al., 2017). We extracted activations from the same eight hidden layers as from the image CNN, in response to all the videos from our stimulus set. Before extracting the activations, we resized each video frame such that its smaller side was 128 pixels, and applied a center crop to obtain 112 × 112 pixels. Pixel values were then normalized by subtracting the ImageNet mean and dividing by the ImageNet standard deviation for each channel. Then, we fed these preprocessed frames to the CNN and extracted the activations from the last layers of its eight residual blocks (the same ones as in the image CNN) using Torch FX in PyTorch, obtaining activations from eight layers in response to the videos from our training, validation, and test sets of videos.

The second step was to prepare the extracted CNN activations for encoding. We flattened the activations for each layer of the image and video CNN across all feature map dimensions, obtaining a 2D matrix of size number of images × number of CNN features. To improve computational efficiency, we reduced the dimensionality of the flattened activations by applying PCA with the number of components that explained at least 90% of the variance in the training set (see Supplementary Table S3 for the number of components), separately for each layer of each image and video CNN. We fitted PCA on the training set activations and projected the training, validation, and test sets onto the resulting components. This yielded CNN activations for eight layers for all stimuli from the training, validation, and test sets, suitably formatted for further encoding analyses.

#### 2.6.3 Predicting the EEG responses from ground-truth annotations

**Fig. 3.**
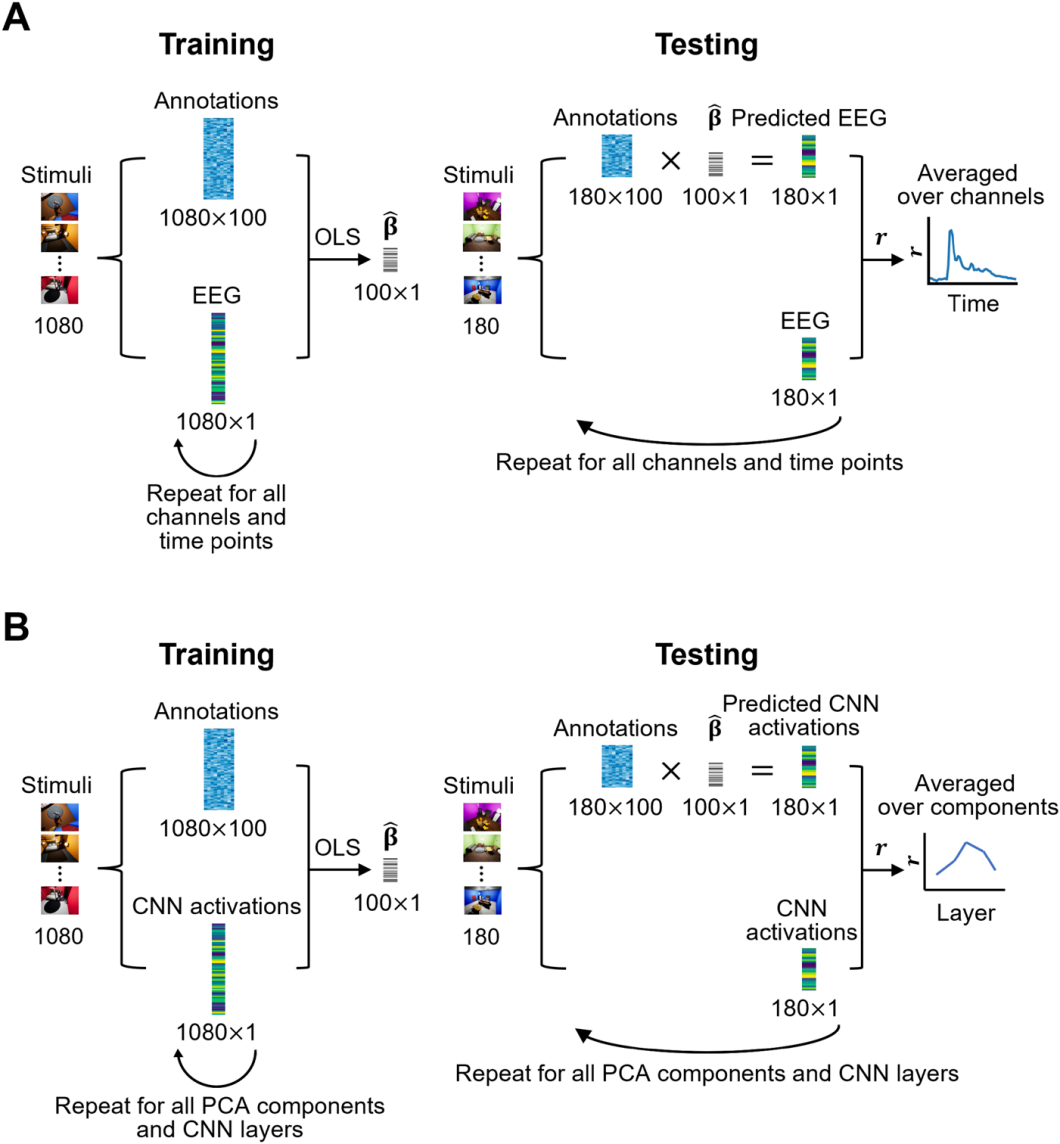
Encoding analysis pipeline. (A) EEG encoding pipeline for one feature. The training set of stimuli and annotations is used to estimate the regression weights β of the regression model, where the annotations predict EEG responses at every channel and time point. Afterward, the test set of stimuli and annotations is used to predict EEG responses from annotations with the estimated regression weights at every channel and time point. The predicted EEG responses are then correlated with the true EEG responses to assess the accuracy of the encoding model at every time point and channel, and the final time course depicts the average correlation over channels. (B) CNN encoding pipeline for one feature. The training set of stimuli and annotations is used to estimate the regression weights β of the regression model, where the annotations predict CNN activations at every principal component and layer. Afterwards, the test set of stimuli and annotations is used to predict CNN activations from annotations with the estimated regression weights at every principal component and layer. The predicted CNN activations are then correlated with the true CNN activations to assess the accuracy of the encoding model at every layer and principal component, and the final layer-wise plot depicts the average correlation over principal components.

To determine when the candidate low-, mid-, and high-level features are processed in the brain, we trained and evaluated linearized encoding models on EEG responses (Fig. 3A; Naselaris et al., 2011). For each subject, feature, EEG channel, and time point, we trained multivariate ridge regression models to estimate the β regression weights from the ground-truth annotations. Ridge regression introduces a regularization hyperparameter, λ, which controls the strength of the L2 penalty on the regression weights, thereby balancing the bias-variance tradeoff.

To optimize λ, we first estimated the β regression weights using ordinary least squares (OLS; Hastie, 2020) and evaluated 30 logarithmically spaced values of λ from $10^{-5}\;$ to $10^{15}$ . Each model’s accuracy was assessed on a validation set by computing the Pearson correlation between predicted and true EEG responses, and the value of λ that maximized encoding accuracy, averaged across channels and time points, was selected as optimal for each feature and subject.

Using the optimized λ, we trained the encoding models on the training set and predicted the EEG responses from the annotations in the test set. We then computed the Pearson correlation between the predicted and true EEG responses. Finally, we averaged the correlation coefficients across channels and subjects, yielding one time course for each low-, mid-, and high-level feature for the image and video conditions.

#### 2.6.4 Predicting CNN activations from ground-truth annotations

To determine which CNN layers capture the candidate low-, mid-, and high-level features, we trained and evaluated linearized encoding models on the dimensionality-reduced CNN activations (i.e., principal components; Fig. 3B). The models predicted these activations from the ground-truth annotations, treating the CNNs as a model of the human visual system. For this, we trained multivariate ridge regression models (akin to the EEG encoding analysis) on flattened and dimensionality-reduced image and video CNN activations separately, for every feature, layer, and principal component, and evaluated them by predicting the respective test set activations and correlating the predicted and true activations for every feature, layer, and principal component. In order to account for the different levels of variance explained by every component, we then calculated, for every feature and layer, the weighted average of the component-specific correlations. In detail, we weighed each component’s correlation by its amount of explained variance, summed the weighted correlations, and divided the sum by the total variance explained. We implemented this approach to assign greater weights to components that contribute more to the overall variance, ensuring that the final correlation results reflect the most informative components rather than being equally influenced by all components. This resulted in layer-wise correlations characterizing the processing of low-, mid-, and high-level features in image and video CNNs.

#### 2.6.5 EEG noise ceiling

To estimate the theoretical maximum encoding accuracy given the level of noise in our EEG responses, we calculated the lower and upper bound of the noise ceiling (Gifford et al., 2022). We computed noise ceiling estimates for every subject and for image and video EEG responses separately. To calculate the lower bound of the noise ceiling, we split all the trials into two equal groups and correlated the preprocessed channel-wise, time point-wise EEG responses between the two groups. To calculate the upper bound of the noise ceiling, we correlated a group containing half of all trials with a group containing all trials. We repeated the calculation of the lower and upper noise ceilings 100 times to permute the grouping of the trials each time and averaged over the permutations and subjects to obtain the final lower and upper noise ceilings.

#### 2.6.6 Correlation between EEG peak latencies and CNN peak layers

To determine whether low-, mid-, and high-level features are processed in a similar hierarchy between the human brain and CNNs, we correlated the EEG peak latencies with the CCN peak layers. For this, we first collected the encoding peak latencies for every one of the 7 features for the EEG and the peak layers from the CNN analyses, for images and videos separately. This resulted in four 7 × 1 vectors. Then, for images and videos independently, we calculated the Spearman correlation between the EEG and CNN vectors, revealing the relationship between the processing hierarchies of the human brain and CNNs.

### 2.7 Statistical analysis

To determine the statistical significance of the decoding and encoding results, we used non-parametric permutation tests, performed separately for each EEG time point and CNN layer. To assess significance for the image and video conditions separately, we performed sign tests with 10,000 permutation samples. For EEG decoding and encoding, each permutation was generated by multiplying the subject-level accuracies by a random vector of 1 and -1 for each time point and recomputing the averaged Pearson correlation across subjects. For CNN encoding, the same procedure was applied to the encoding accuracies for each principal component at each layer. To assess the significance of differences between image and video conditions, we performed a permutation test by randomly swapping the condition labels across subjects (EEG) and principal components (CNN) 10,000 times. The Pearson correlation was recomputed and averaged across subjects or principal components, respectively. For both tests, we obtained the p-value of the observed data by calculating the rank of its mean with respect to the distribution of the permutation samples. Finally, we controlled for multiple comparisons by adjusting the p-values of the original data for their inflated false discovery rate (FDR) using the Benjamini–Hochberg procedure (Benjamini & Hochberg, 1995) with α = 0.05 (two-tailed).

Additionally, we calculated the bootstrapped 95% confidence intervals (CIs) for the mean EEG decoding and encoding accuracies across subjects to estimate the uncertainty of these sample means. To estimate the bootstrapped distributions, we resampled subject-level accuracies 10,000 times for each EEG decoding and feature-specific image and video encoding time course, and computed the 95% CIs (2.5th and 97.5th percentiles) from these distributions. To provide a comparable measure for the CNNs, we applied the same procedure to the CNN encoding results. For each layer, we resampled the encoding accuracies of its principal components 10,000 times and calculated the 95% CIs of the resulting bootstrapped distributions. These intervals reflect the variability of the mean encoding accuracy across principal components and are intended to provide an analogous measure of uncertainty for the CNN encoding results.

Moreover, we calculated the bootstrapped 95% CIs for the peak latencies of EEG encoding time courses and peak layers of the CNN encoding results. We used these CIs to (1) estimate the uncertainty around our sample peak latencies and layers and to (2) test whether the differences between the encoding peak latencies and layers for the different features were statistically significant. To address the latter, for each pairwise combination of features from the image and video results separately, we created the bootstrapped distribution for the difference between peaks, and whenever the 95% CI for the difference between two conditions contained 0, the difference was deemed non-significant.

To determine the significance of the EEG peak latency and CNN peak layer correlation, we permuted the feature-specific CNN peak layers (resulting in 5040 permutations) and correlated, for each permutation, the shuffled vector of peak layers with the true EEG peak latencies. This created a distribution of sample correlations based on which we determined whether the original correlation was significant (i.e., if its p-value was less than or equal to 0.05).

## 3 Results

### 3.1 Decoding images and videos from EEG yields comparable results

To test whether EEG signal quality was comparable for images and videos, we performed time-resolved pairwise decoding (Cichy et al., 2014; Grootswagers et al., 2017). Given that video data might be noisier due to more frequent eye movements (Böhme et al., 2006), we compared the decoding time courses for image- and video-evoked responses.

**Fig. 4.**
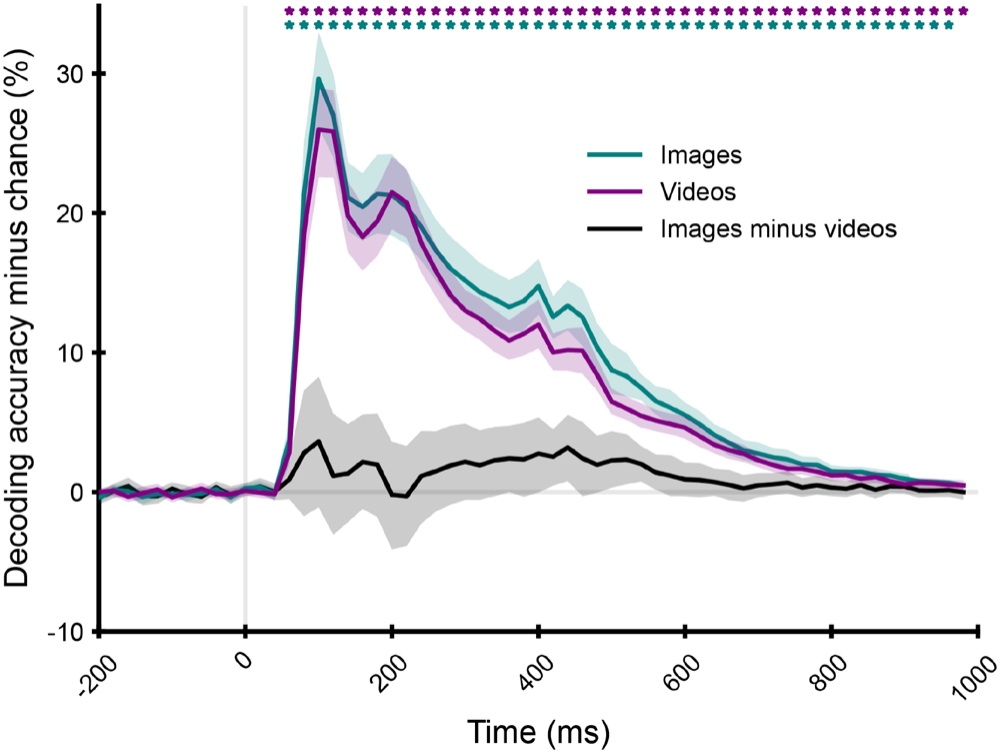
Decoding scene identity from images and videos. Averaged pairwise decoding results for individual scene images (15 subjects, green curve) and videos (20 subjects, purple curve). The difference curve (images minus videos) is shown in black. We indicated the significant time points (two-tailed, p ≤ 0.05, FDR-corrected) with stars above the time course, the 95% confidence intervals with shaded areas around the curves, the stimulus onset with a vertical gray line, and the decoding chance level with a horizontal gray line.

We observed similar time courses for image and video decoding (Fig. 4). For both, we identified an onset of decoding at 60 ms after stimulus onset (p ≤ 0.05, FDR-corrected), meaning that individual scenes were distinguishable in the brain starting from then. In both cases, decoding was significant (p ≤ 0.05) throughout most of the trial and peaked at 100 ms ([95% CI]: images: [100 ms, 100 ms], videos: [100 ms, 120 ms]) after stimulus onset, showing that individual scenes were best distinguishable at that time. The difference time course between image and video decoding was not significant at any time point, further suggesting a similar quality of the signal between image and video EEG responses. Overall, the decoding results show that scene information is as well decodable from image as from video EEG responses, suggesting that the quality of the signal is comparable between the two datasets. This indicates that the two datasets can be analyzed using equivalent methods and that any observed differences are not solely due to data quality.

### 3.2 Candidate mid-level features are most strongly encoded in the brain between ~100 and ~250 ms after stimulus onset

To determine the time course of mid-level feature processing in the brain with respect to low- and high-level features, we trained and evaluated encoding models that predicted EEG responses from ground-truth annotations of one low-level feature (edges), five candidate mid-level features (reflectance, lighting, world normals, scene depth, and skeleton position), and one high-level feature (action identity). We performed the encoding analysis on image and video responses separately and compared the encoding time courses to assess how motion information affects mid-level feature processing.

**Fig. 5.**
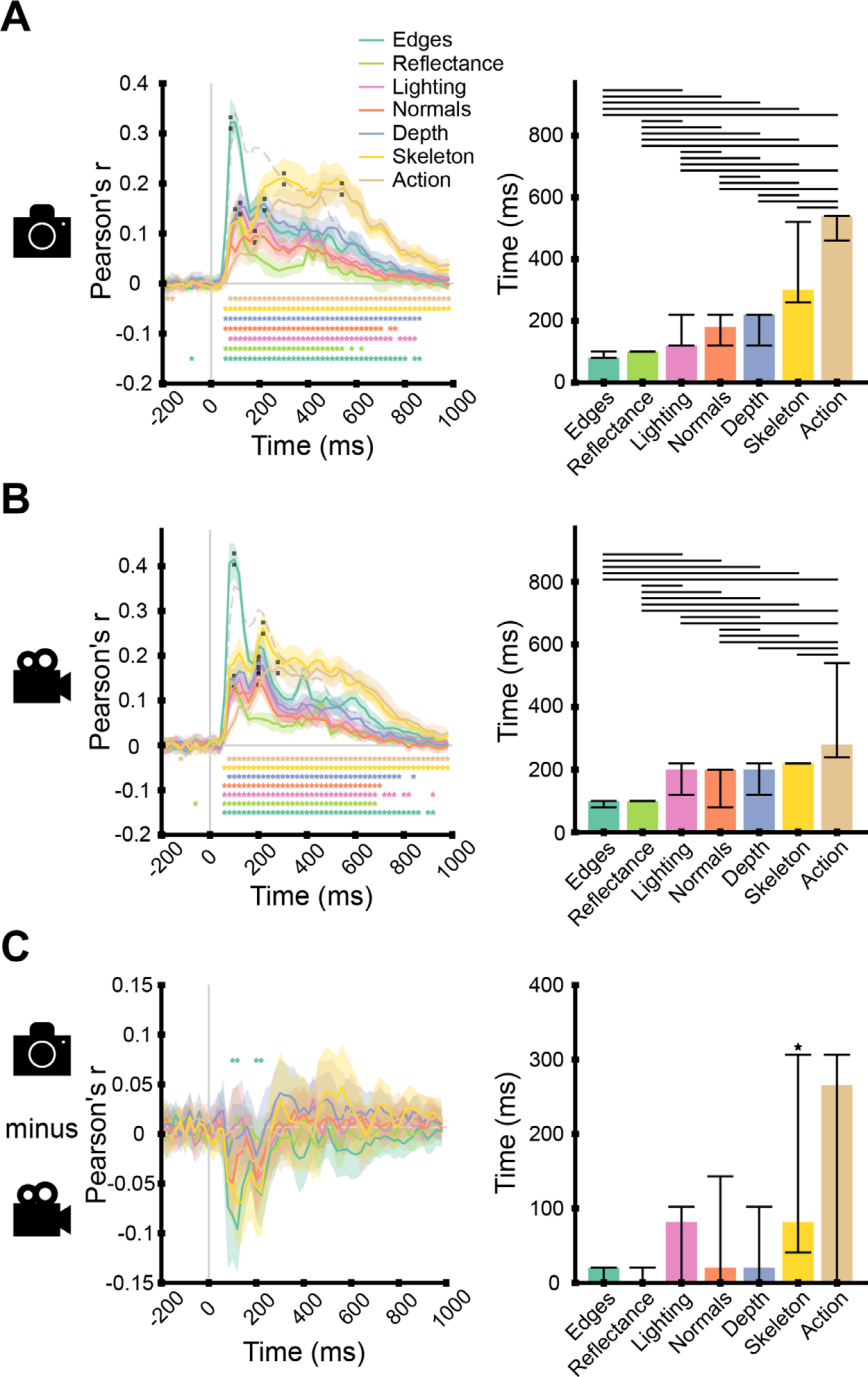
Predicting low-, mid-, and high-level feature EEG representations in scene images and videos from their ground-truth annotations. Encoding time courses for EEG responses collected during the viewing of (A) images (15 subjects), (B) videos (20 subjects), and (C) their difference. Left panel: the time course of one low-, five mid-, and one high-level feature representations. We indicated the significant time points with stars (two-tailed, p ≤ 0.05, FDR-corrected), the 95% confidence intervals with shaded areas around the curves, the stimulus onset with a vertical gray line, the chance level with a horizontal gray line, the lower noise ceiling with a dashed gray line (see Supplementary Note S10 and Supplementary Fig. S12 for upper noise ceilings), and the peak latencies with short dashed lines. Right panel: peak latencies of the feature-specific encoding curves. In (C), the right plot represents the differences in peak latencies between images and videos. We indicated the 95% confidence intervals with vertical error bars, the significance (p ≤ 0.05) between feature peak latencies with horizontal bars, and the significant peak latency differences with stars above the error bars.

We made three key observations. First, for both image and video data, the ground-truth annotations for all low-, mid-, and high-level features predicted the EEG responses significantly from ~60 ms on (p ≤ 0.05, FDR-corrected) throughout most of the trial (Fig. 5A and B, left). This demonstrates that all feature annotations capture information that is reflected in the EEG responses to our stimulus set. For video data, we observed very similar results when using the ground-truth annotations from the first frame or from the last frame of the videos (see Supplementary Note S3 and Supplementary Fig. S2), aligning with the high similarity of representations across video frames (see Supplementary Fig. S3), and further supporting the robustness of our results.

Second, we observed that the encoding accuracy for our candidate mid-level features peaked between ~100 and ~250 ms after stimulus onset, for both images and videos (Fig. 5A and B, right). Of the mid-level features, for both images and videos, the encoding accuracy peaked first for reflectance and last for skeleton position, while the results for lighting, world normals, and scene depth were in between them and peaked close to one another (see Supplementary Table S2 for statistical details). For both images and videos, the results for all mid-level features except for reflectance peaked significantly after the low-level feature, that is, edges (on average, 94 ms later, significant differences indicated by lines above bar plots), and significantly before the high-level feature, that is, action identity (on average, 226 ms earlier). This further supports our choice of candidate mid-level features and aligns with classical hierarchical frameworks (Biederman, 1987; Marr, 1982; Riesenhuber & Poggio, 1999).

Third, there were almost no significant differences in the encoding accuracy between images and videos except for four time points in the edges time course between 100 and 220 ms (Fig. 5C, left). The similarity between image and video results is partly explained by the similarity of ground-truth annotations, which are highly correlated (see Supplementary Note S5 and Supplementary Fig. S5), and when used interchangeably (i.e., using image annotations to predict EEG responses to videos), produce comparable results (see Supplementary Fig. S6). However, the peak latency differed for one mid-level feature, skeleton position, exhibiting a later peak in images than in videos (difference between images and videos: 80 ms, 95% CI [40, 300]). The earlier latency for videos may reflect facilitated extraction of motion-related features (Isik et al., 2018; Johansson, 1973). This finding is corroborated by a time-generalization analysis (Cichy et al., 2014; King & Dehaene, 2014), in which encoding models trained at each time point were tested across all other time points. The results revealed a pattern consistent with delayed, rather than sustained, processing for images (see Supplementary Note S2 and Supplementary Fig. S1). However, latency differences decreased when task-relevant trials were excluded (see Supplementary Note S4 and Supplementary Fig. S4), suggesting that the later peak for image responses could reflect task-dependent activity rather than a fundamental processing delay.

Overall, our candidate mid-level features were processed between ~100 and ~250 ms after stimulus onset, that is, between low- and high-level features, for both images and videos. One mid-level feature related to biological motion, skeleton, showed an earlier peak latency in videos, and except for a few time points in the edges time course, encoding accuracy did not differ significantly between stimulus types.

### 3.3 Candidate mid-level features are processed in CNNs throughout the layer hierarchy

To determine whether CNNs as a model of the human visual system encode the candidate mid-level features in a similar way to humans, we first trained and evaluated encoding models that predicted CNN layer activations during stimulus presentation from ground-truth annotations, separately for images and videos. For this, we selected one image CNN pre-trained on scene classification with images (ResNet-18; He et al., 2015; Zhou et al., 2018) and one video CNN pre-trained on action classification with videos (3D ResNet-18; He et al., 2015; Tran et al., 2018) and collected their activations from eight layers in response to the image and video stimuli, respectively. We then trained encoding models to predict their activations to the training set stimuli from ground-truth annotations. Then, for each network, layer, and feature, we applied the trained encoding models to predict the activations to the test set stimuli from ground-truth annotations. Lastly, we calculated the encoding accuracy by correlating the predicted and the true activations for every network, layer, and feature, thereby characterizing the layer-wise processing of candidate low-, mid-, and high-level features for image and video CNNs.

**Fig. 6.**
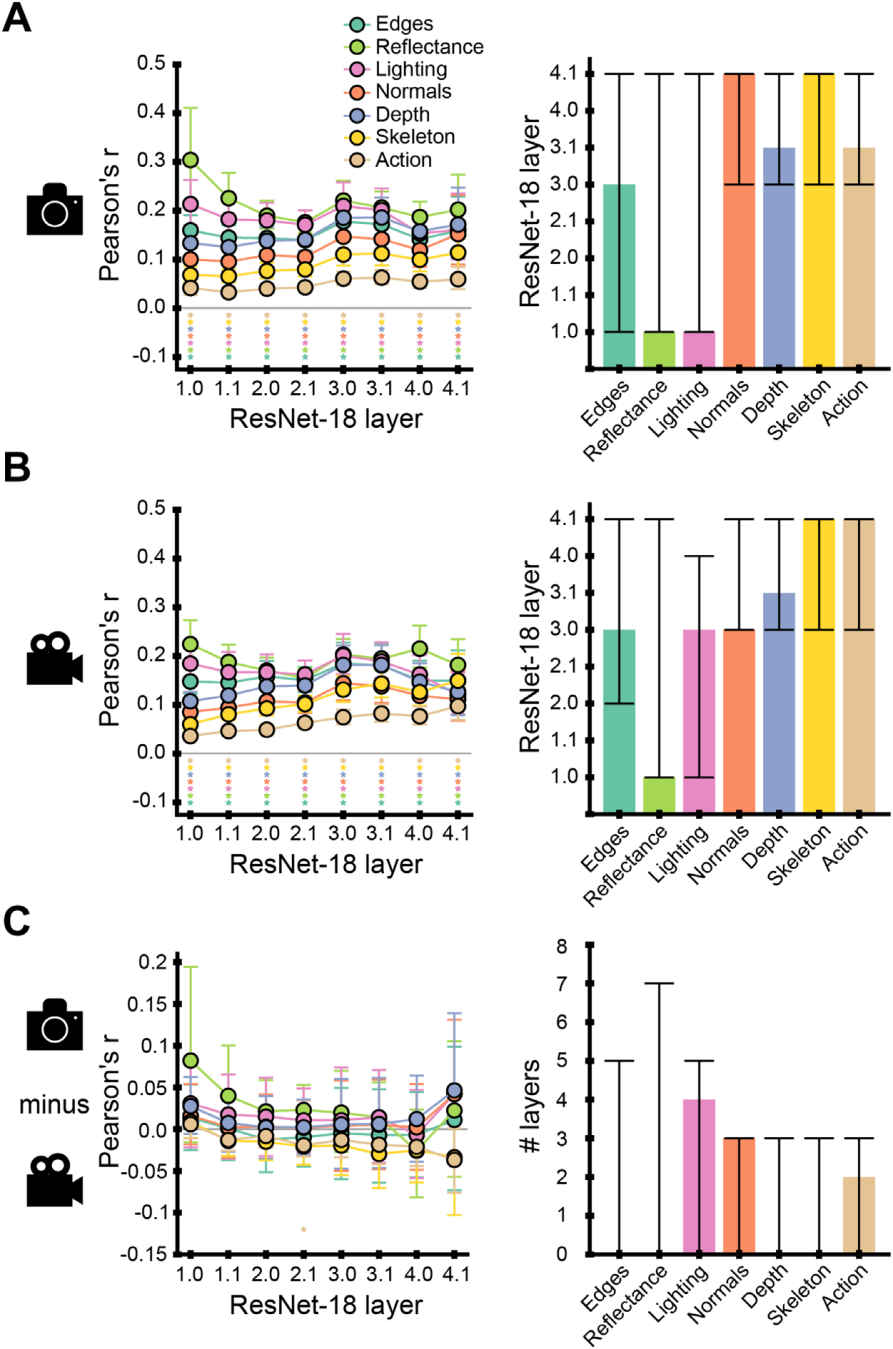
Predicting low-, mid-, and high-level feature CNN representations in scene images and videos from their ground-truth annotations. Encoding results for CNN activations collected during the presentation of (A) images to ResNet-18 trained on scene classification, (B) videos to 3D ResNet-18 trained on action classification, and (C) their difference. Left panel: encoding accuracy for one low-, five mid-, and one high-level feature representations, for eight CNN layers (named 1.0, 1.1, 2.0, 2.1, 3.0, 3.1, 4.0, and 4.1). We indicated the peak layers with dashed lines, the significant layers with stars (two-tailed, p ≤ 0.05, FDR-corrected) and the 95% confidence intervals with shaded areas around the curves. Right panel: peak layers of the encoding accuracies for the seven features. In (C), the right plot represents the differences in peak layers between images and videos. We indicated the 95% confidence intervals with vertical error bars and the significance (p ≤ 0.05) between feature peak layers with horizontal bars.

We made two main observations. First, for both image and video CNNs, the ground-truth annotations predicted significantly (p ≤ 0.05, FDR-corrected) the activations for all features and layers (Fig. 6A and B, left, significance indicated by stars). This indicates that the annotations capture information represented in the CNN activations.

Second, the ground-truth annotations of our candidate mid-level features significantly predicted activations across multiple CNN layers, from the first to the penultimate layer. However, peak encoding accuracy did not consistently occur at intermediate layers. While encoding of scene depth peaked at intermediate layers in both CNNs (layer 3.1), the encoding of other mid-level features, such as reflectance and lighting, peaked very early (layer 1.0 for the image CNN; layers 1.0 and 3.0 for the video CNN). Conversely, the encoding of some candidate mid-level features peaked in late layers, such as world normals in the image CNN (layer 4.1) or skeleton position in both CNNs (layer 4.1). Wide 95% confidence intervals around these peaks further suggest a less pronounced, shallower hierarchy (Fig. 6, right panel). Control analyses indicate that the feature processing hierarchy depends in part on (1) the training objective, whereby a CNN trained on action classification with images shows a qualitatively different feature processing hierarchy than a CNN trained on scene classification with images (see Supplementary Note S6 and Supplementary Figs. S7 and S8) and (2) the input resolution (see Supplementary Note S7 and Supplementary Fig. S9). Together, these control analyses underscore the importance of systematic comparisons across different network design choices and suggest that low-, mid-, and high-level feature processing is sensitive to factors such as training objective and input resolution.

To summarize, our encoding analysis results show that candidate mid-level features are represented across multiple CNN layers with a shallower and less consistent hierarchy than in humans.

### 3.4 The processing hierarchy of candidate mid-level features correlates between humans and CNNs only for videos

After characterizing how low-, mid-, and high-level features are encoded across CNN layers, we next assessed whether the human brain and CNNs process them via a similar hierarchy. To test this, we compared the encoding peak latencies in EEG with the peak layers in CNNs for all features, separately for images and videos, both qualitatively and quantitatively using Spearman’s correlation.

We first observed qualitatively that ground-truth annotations predicted the CNN activations for candidate mid-level features, but not for low- or high-level features, in a similar order as in the EEG results. For videos, in both the human brain and CNNs, the earliest represented mid-level features were reflectance and lighting, followed by world normals, then scene depth, and finally, skeleton position. For images, the similarity was less pronounced: while for both humans and CNNs the earliest represented mid-level features were reflectance and lighting, in humans, this was followed by world normals then scene depth, and finally, skeleton position, and in CNNs, world normals were processed at the same time as skeleton position, that is, latest of all. The low-level feature (edges) was predicted in humans before any mid-level feature, while in CNNs, it was predicted between mid-level features. The high-level feature (action identity) was represented in humans after all mid-level features, while in CNNs, it was represented at the same time as some mid-level features. Therefore, unlike in EEG, where edges were processed first and action identity was processed last, in CNNs, edges and action identity were processed synchronously with mid-level features.

**Fig. 7.**
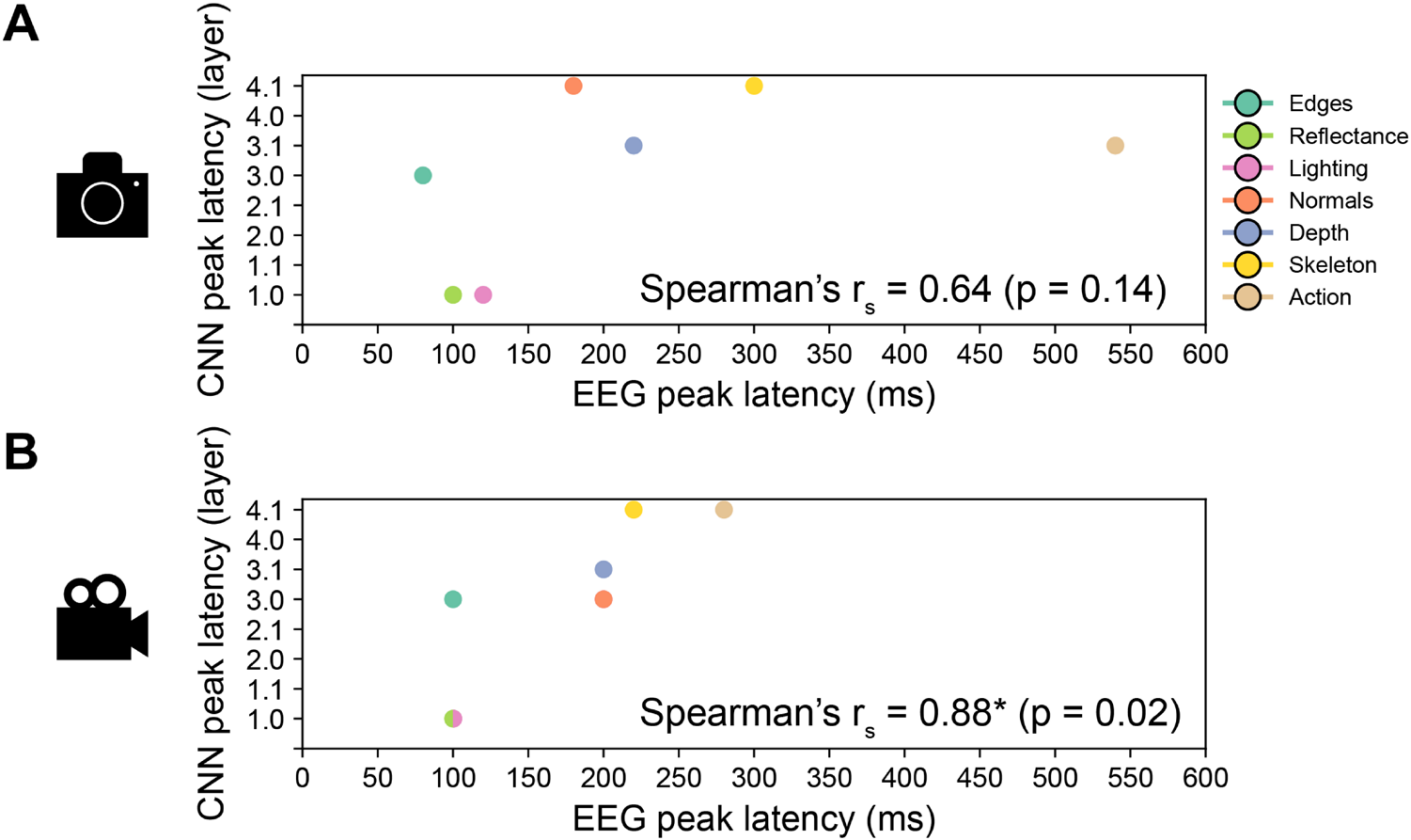
Correlating EEG encoding peak latencies and CNN encoding peak layers. Results for the Spearman’s correlation between the EEG and CNN encoding peak latencies for the analysis on (A) images and (B) videos. We indicated the Spearman’s coefficient value and its significance (p ≤ 0.05).

To quantify these observations, we calculated the Spearman’s correlation coefficient, *r_s_,* between EEG peak latencies and CNN peak layers, for images (Fig. 7A) and videos (Fig. 7B). In line with our qualitative description, we observed that the correlation was significant (p ≤ 0.05) for videos (*r_s_* = 0.88, p = 0.02), but not for images (*r_s_* = 0.64, p = 0.14). We further calculated the correlation only for mid-level features, and consistent with the results for all features, observed that the correlation was significant for videos (*r_s_* = 0.92, p = 0.01) but not for images (*r_s_* = 0.79, p = 0.14). Together, this points to similarities in the processing order of scene features in humans and CNNs, particularly for mid-level feature processing during video viewing. However, since CNN encoding accuracy peaks depend on the training objective and other design choices, and because our peak latency/peak layer correlations aggregate signals across units, stimuli, and subjects, these results should be interpreted cautiously. They represent a coarse, abstract characterization rather than a definitive mapping between human and CNN processing.

Overall, these results suggest that mid-level features, but not low- or high-level features, are processed with a similar hierarchy in the human brain and artificial neural networks during the viewing of videos.

## 4 Discussion

### 4.1 Summary of results

We introduced a novel stimulus set of naturalistic 3D-rendered images and videos with ground-truth annotations of five candidate mid-level features to investigate mid-level vision in humans and in models of the human visual brain, CNNs. We report two main findings. First, candidate mid-level features were most strongly represented in the brain between ~100 and ~250 ms after stimulus onset, between low- (90 ms) and high-level (410 ms) features. Second, although CNNs showed a shallower and less consistent hierarchy, mid-level features, but not low- or high-level features, were processed in a similar relative order to that observed in the human brain during video presentation.

### 4.2 Candidate mid-level features are represented between low- and high-level features in the human brain

Leveraging a novel stimulus set with ground-truth annotations and an exploratory, data-driven approach, we showed that candidate shape- and surface-related mid-level features were best represented between ~100 and ~250 ms post-stimulus. This aligns with previous reports that mid-level feature information can be extracted from objects between 100 and 400 ms (Grootswagers et al., 2019; Proklova et al., 2019; Wang et al., 2022). Given that low-level information such as edges and luminance is processed in the cortex as early as at 50 ms (Cichy et al., 2014; Di Russo et al., 2002), and high-level semantic information such as animacy (Carlson et al., 2013; Cichy et al., 2014) or action identity (Isik et al., 2018) is best represented at 200–400 ms and later, this places our candidate mid-level features right between low- and high-level features.

While the classical hierarchical framework is a simplification, and is partially inaccurate in the context of scene perception (Groen et al., 2017), it remains valuable from a pragmatic perspective. Like many scientific models, it functions as an abstraction that highlights certain aspects of the target system while misrepresenting others, enabling researchers to draw inferences, generate predictions, and deepen understanding (Gelfert, 2016). This framework has guided decades of research in visual perception (Biederman, 1987; Marr, 1982; see also Epstein & Baker, 2019; Groen et al., 2017; Peirce, 2015), shaped the development of computer vision (Forsyth & Ponce, 2003; LeCun et al., 2015), and continues to provide a tractable tool for interpreting complex and opaque representational spaces in both biological and artificial neural networks. Consistent with classical hierarchical theories of vision (Biederman, 1987; Marr, 1982; Riesenhuber & Poggio, 1999), our findings reinforce the role of mid-level features in bridging low- and high-level vision.

We take our findings to be facilitated by four advances of our experimental approach. First, our stimulus set was composed of 3D scenes that depicted characters performing diverse actions in realistic environments, offering richer and more naturalistic inputs than objects on uniform backgrounds. This approach enabled us to study mid-level processing under conditions closer to everyday vision (Felsen & Dan, 2005), integrating scene, object, and action perception.

Second, using a stimulus set with ground-truth annotations allowed us to link stimulus features and time courses of neural processing with high precision. Most previous studies relied on deep neural network-based approximations of mid-level features, such as Taskonomy outputs (Zamir et al., 2018) or on data-driven CNN activations (Kubilius et al., 2016) that are noisier and more difficult to interpret semantically. In contrast, ground-truth annotations address these two shortcomings by depicting the noise-free true state and by capturing interpretable mid-level features.

Third, our stimulus set was annotated for multiple candidate mid-level features, aligning with empirical evidence linking mid-level processing to surface and shape representations (e.g., Lescroart & Gallant (2019) for a similar approach using fMRI data; Choo & Walther, 2016; Pasupathy & Connor, 1999, 2001). Although far from exhaustive, it allowed us to capture the diverse nature of mid-level features more comprehensively than studies assessing fewer features (Beeck et al., 2008; Jagadeesh & Gardner, 2022; Yue et al., 2020), and on level ground in a unified experimental setting.

Fourth, our approach characterized the mid-level feature processing in both still images and dynamic videos. Given that motion impairments can affect shape perception (Scholte & Haan, 2025) and anterior subdivisions of scene-selective regions, previously linked to mid-level features, are especially sensitive to dynamic stimuli (Çukur et al., 2016), we expected to find differences across those processing formats. Surprisingly, except for the timings observed for skeleton position, motion information in videos did not affect the processing hierarchy, likely because image and video annotations were highly similar. We view detailed analyses of camera viewpoint or related stimulus manipulations as an interesting direction for future work, which could reveal how these factors modulate static and dynamic feature processing.

### 4.3 Candidate mid-level features, but not low- or high-level features, are processed via a similar hierarchy in humans and CNNs during video presentation

As a proof of concept, we compared the hierarchies of feature processing of humans with those of image-computable models to determine their suitability as brain models in this regard. We made two observations.

First, we observed that mid-level feature processing in videos follows a similar hierarchy in CNNs and humans. This aligns with prior studies demonstrating hierarchical correspondence between CNNs and the visual cortex: intermediate layers best predict mid-level regions, while later layers best predict high-level regions (Cichy et al., 2016; Yamins et al., 2014). Our work builds on these studies but takes a complementary approach. Instead of using CNN activations to predict brain data, we used ground-truth annotations of candidate mid-level features to predict CNN activations. This provides a new perspective on what these layers encode and how they align hierarchically with the brain. Our results support previously observed similarities in the nature and processing stage of mid-level representations in CNNs and humans. For instance, Kubilius et al. (2016) reported correlations between intermediate object-trained CNN layer activations and human perceptual similarity judgments of shape, which in turn correlated with neural activity in V3 and lateral occipital cortex (Beeck et al., 2008). However, we note that the results in the current study only pertain to dynamic stimuli, and that the hierarchy observed in CNNs was shallower and less robust than in the human brain, pointing to existing discrepancies between humans and CNNs (Sexton & Love, 2022).

Second, we found that there is a deviation in hierarchical correspondence in terms of our framing features. For one, we observed that edge processing occurs at different latencies in humans and CNNs, which we attribute to our choice of edge detection algorithm, canny edges. While early CNN layers are often described as edge detectors (LeCun et al., 2015), they likely do not approximate the Canny algorithm to extract edge information (Canny, 1986). Instead, canny edge outputs resemble silhouettes and potentially engage shape-extracting mechanisms later in the CNN hierarchy. Since they share similarities with human drawings (Borji & Itti, 2014), which are processed early in humans (Singer et al., 2023) but in intermediate CNN layers (Singer et al., 2022), this latency difference between CNNs and humans is plausible. Moreover, we observed that action is represented at different stages of processing in humans and CNNs for images, but not in videos. This likely reflects that the image CNN was trained on scene rather than action classification, in contrast to the video CNN, leading it to represent action in intermediate rather than late layers. These observations align with previous studies reporting a lack of hierarchical correspondence between humans and CNNs (Sexton & Love, 2022; Xu & Vaziri-Pashkam, 2020), emphasizing the need for further research to ascertain whether humans and machines process visual input in similar stages.

### 4.4 Limitations and future directions

In this work, given the lack of a fully stabilized conceptual definition of mid-level features, we selected a candidate set of mid-level features as a testing ground for the investigation of mid-level feature processing in humans and CNNs. We highlight two limitations to our approach. First, our selected features are not independent and are not processed discretely: in fact, we observed overlaps between several annotations (see Supplementary Note S8 and Supplementary Fig. S10 for representational similarity analysis (RSA) and centered kernel alignment (CKA) results showing similarities between annotations for different features). For example, we observed similarities between world normals and scene depth, lighting and reflectance, and skeleton position and action identity, suggesting that they captured related information. The brain likely exploits these regularities for computational efficiency (Scholte & Haan, 2025), as reflected in the supplementary variance partitioning analysis results (see Supplementary Note S9 and Supplementary Fig. S11). This suggests that alternative decompositions might achieve comparable or better encoding performance.

Second, our results depend strongly on CNN design choices, including training objectives and preprocessing. Thus, our findings serve as a proof of concept, showing how ground-truth annotations from 3D-rendered scenes can probe feature processing in artificial neural networks, though further work is needed to assess how design choices affect hierarchical alignment.

## 5 Conclusion

Using a novel stimulus set with ground-truth annotations from naturalistic 3D-rendered still and dynamic scenes, we showed that surface- and shape-related mid-level features are processed between low- and high-level features in humans (~100–250 ms) and for dynamic scenes, in a similar relative order in CNNs, underscoring the bridging role of mid-level vision between sensory and semantic processing.

## Supplementary Material

Supplementary Material

## Data Availability

The neural data, stimuli, and ground-truth annotations are uploaded on https://osf.io/7c9bz/. The code used for this project is under https://github.com/Agnessa14/Mid-level-features.
