# Supplementary material for: Investigating the temporal dynamics and modeling of mid-level feature representations in humans
Source: Imaging Neurosci (Camb). 2026 Apr 27;4:IMAG.a.1207. doi: 10.1162/IMAG.a.1207 (PMC13125056; doi:10.1162/IMAG.a.1207)
Supplement: Supplementary Material [file IMAG.a.1207_supp.pdf]

Supplementary Information

Agnessa Karapetian, Alexander Lenders, Vanshika Bawa, Martin Pflaum, Raphael Leuner, Gemma Roig, Kshitij Dwivedi, Radoslaw M. Cichy

Investigating the temporal dynamics and modelling of mid-level feature representations in humans

Supplementary Note 1. Rendering pipeline

The generation of the 1440 rendered videos was automated. Each video contains exactly 40 frames. Rendering was performed by iteratively playing and pausing the animation, saving the rendered frame, and extracting the rendered visual features (lighting, reflectance, scene depth, world normals and skeleton position). Each video represents a unique combination sampled from one of 20 rooms, one of 4 camera spawn positions, one of 6 actions, and one of 3 characters. The camera spawn positions define locations near which the camera will spawn randomly. The camera rotation was chosen so that it always looks at the character, with additional random shifts ensuring that the character is not always centered in the frame. The rooms themselves specified the positions of the walls, floor, and ceiling, as well as the spawn points for objects and actions within the room. Objects, wall and ceiling paintings, and floor types were chosen randomly; however, they almost never overlapped, as objects were scaled to fit within predefined volumes, and the spawn points were selected to prevent these volumes from intersecting. The same applies to actions, where spawn points were chosen to ensure that characters do not overlap with their surroundings. Often, the spawn points for each action are not unique, allowing an action to be performed at different locations within a room. This approach enabled the rendering of a large variety of videos while ensuring that the scenes appear realistic and free of overlapping objects.

Supplementary Note 2. Time generalization analysis

In the main manuscript, peak latencies for skeleton position were significantly later for images than for videos (**Figure 5C**), which may reflect a facilitative role of dynamic video information in (biological) motion processing (Isik et al., 2018; Johansson, 1973). An alternative explanation is sustained processing for images: the representation may continue to refine over time, producing later peaks without a later onset. Here, sustained processing refers to either prolonged computation (e.g., via recurrence) or extended maintenance of a representation.

To test this, we conducted a time generalization analysis separately for each feature and modality (Cichy et al., 2014; King & Dehaene, 2014). Encoding models were trained on EEG responses at a given time point and tested across all other time points, producing a time generalization matrix where the rows correspond to the training times and the columns correspond to the testing times. If later image peaks reflected sustained processing, diagonals would be broader, indicating persistence over time. As shown in **Supplementary Figure 1**, generalization patterns were similar for images and videos, supporting a delayed rather than sustained processing interpretation.

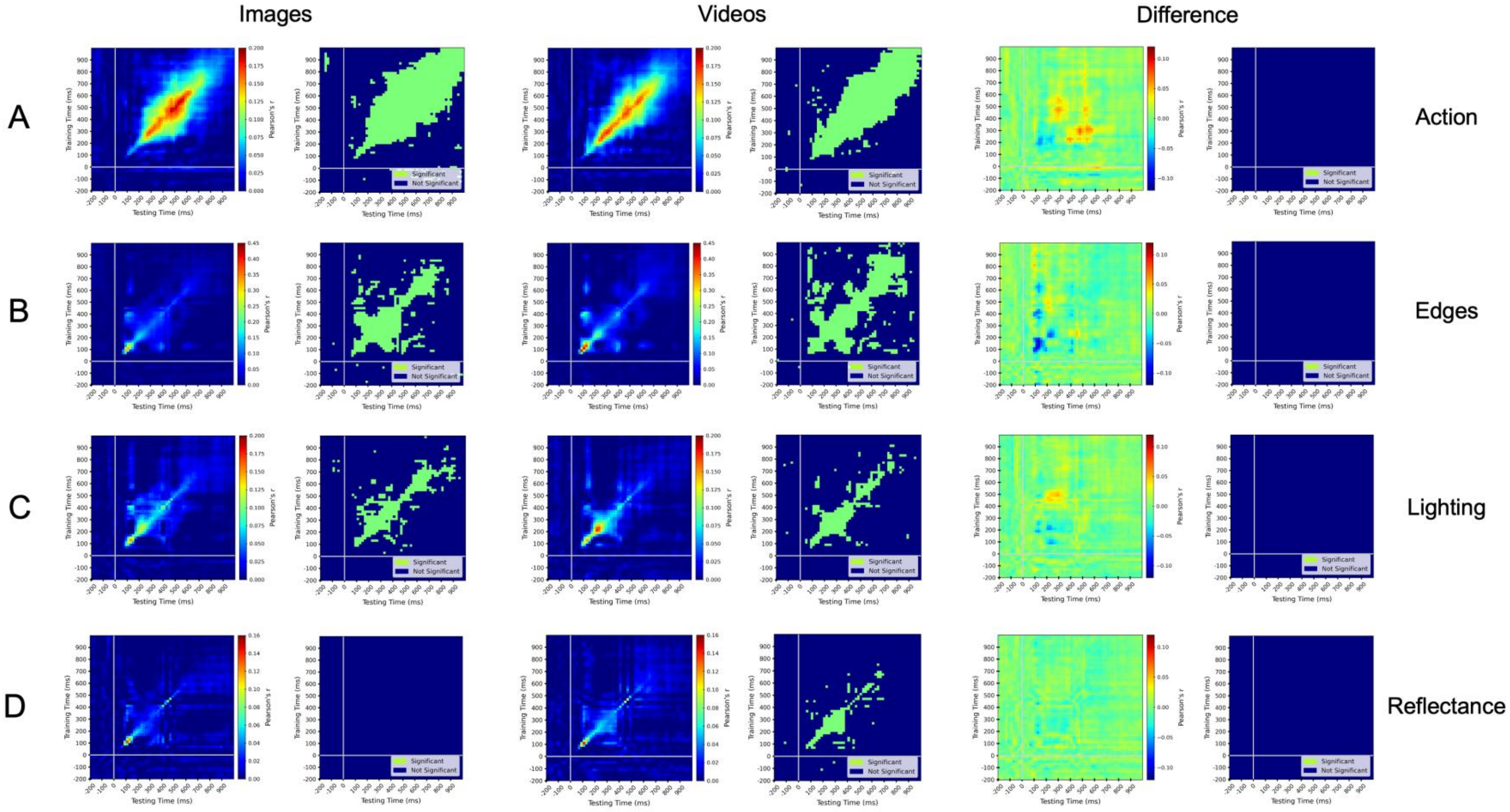
Images
Videos
Difference
A
B
C
D
Action
Edges
Lighting
Reflectance
Training Time (ms)
Testing Time (ms)
Pearson's r
Significant
Not Significant

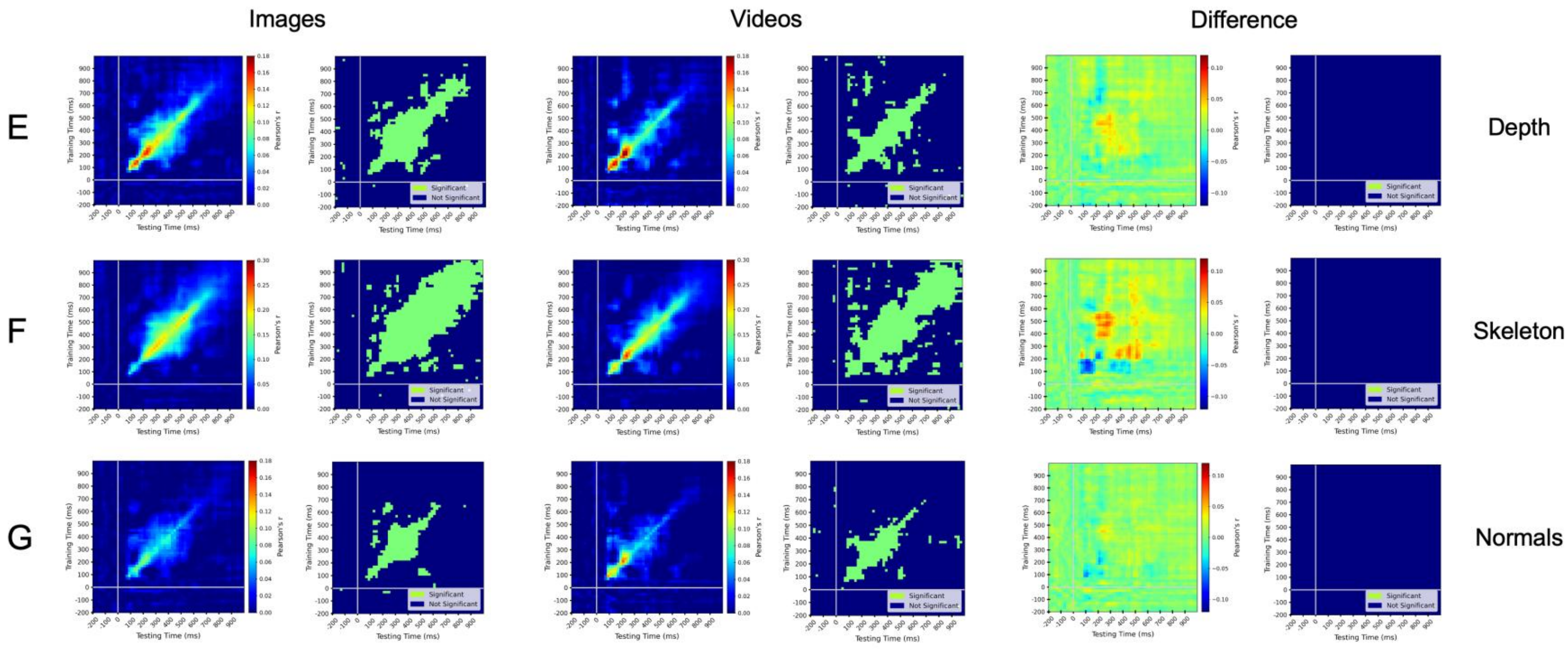

Supplementary Figure 1. Time generalization analysis of EEG encoding models for images and videos. Time generalization matrices show how well encoding models trained to predict EEG responses at a given time point generalize across all other time points (rows represent training time, columns represent testing time). Results are shown for features (**A**) action identity, (**B**) edges, (**C**) lighting, (**D**) reflectance, (**E**) scene depth, (**F**) skeleton position, and (**G**) world normals. Columns 1-2 show EEG data during image viewing (15 subjects), columns 3-4 for video viewing (20 subjects), and columns 5-6 their difference (images minus videos). Matrices represent participant-averaged encoding accuracies. Statistical significance was assessed using two-tailed permutation tests ($p \leq 0.05$, FDR-corrected): within each modality, values were tested against zero via a permutation sign test; differences between image and video conditions were tested by randomly permuting condition labels. Significant time points are marked in green. Across all features, no significant differences in time generalization patterns were observed between images and videos, suggesting that later peak latencies for images likely reflect delayed rather than sustained processing.

Supplementary Note 3. Impact of frame averaging on temporal latencies in video EEG encoding

In the main analysis, ground-truth annotations for videos were averaged across frames (Section 2.6.1). To evaluate whether this averaging affected peak latencies, we repeated the encoding analysis using features from (**a**) only the first frame and (**b**) only the last frame of each video. Apart from slightly lower encoding accuracies for edges, as shown in **Supplementary Figure 2**, frame-specific features produced encoding curves and peak latencies consistent with those in **Figure 5B** (reproduced in **Supplementary Figure 2A**). This robustness aligns with the high similarity of feature representations across video frames (**Supplementary Figure 3**), indicating that successive frames contain largely redundant information.

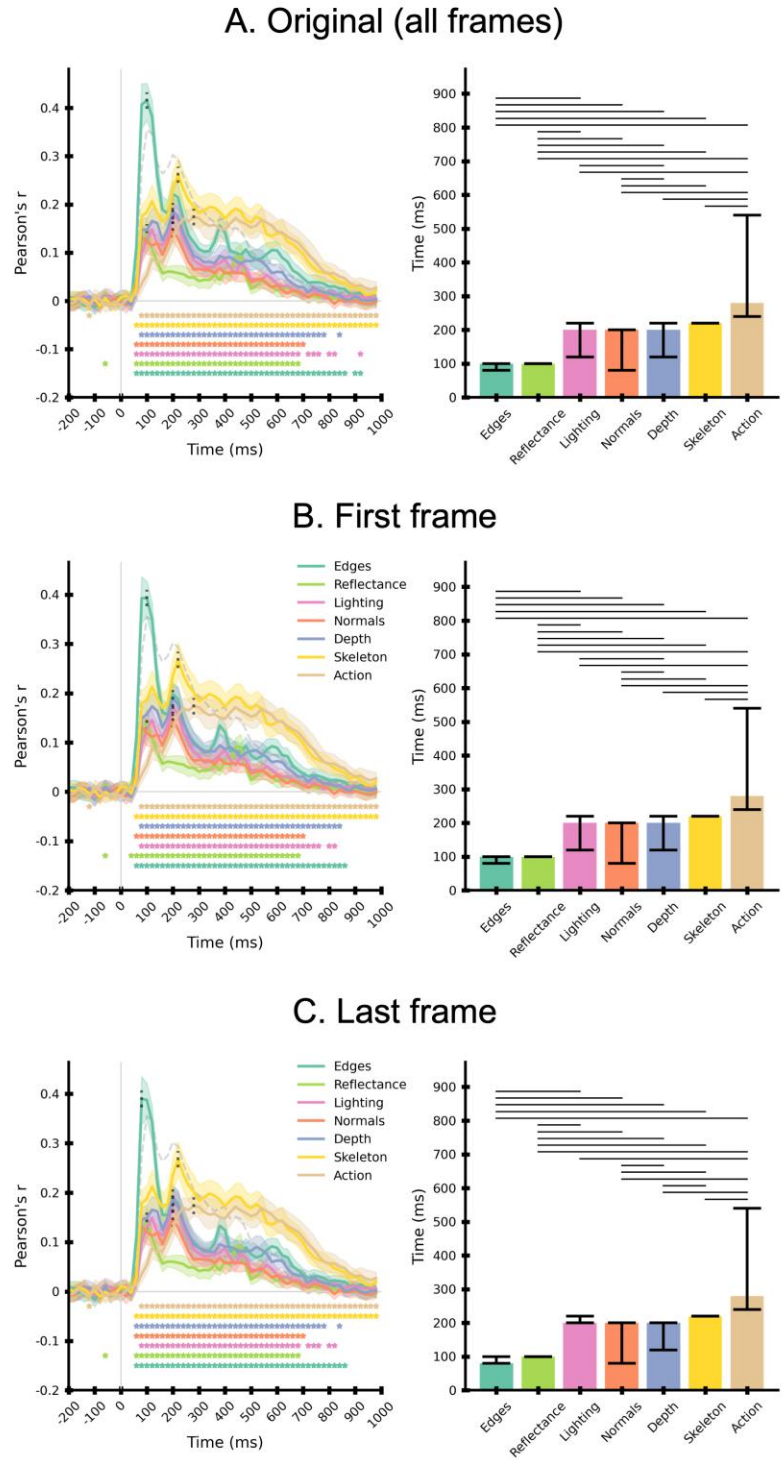

Supplementary Figure 2. Replication of video EEG encoding analysis using frame-specific features. Encoding time courses for the EEG responses predicted from ground-truth annotations extracted from (**A**) all frames and averaged (main analysis), (**B**) only the first frame and (**C**) only the last frame of each video. Left panel: the time course of one low-, five mid-, and one high-level feature representations. We indicated the significant time points with stars (two-tailed, $p<0.05$, FDR-corrected), the 95% confidence intervals with shaded areas around the curves, the stimulus onset with a vertical gray

line, the chance level with a horizontal gray line and the noise ceiling with a shaded gray area. Right panel: peak latencies of the features. We indicated the 95% confidence intervals with vertical error bars, the significance (p≤0.05) between feature peak latencies with horizontal bars, and the significant peak latency differences with stars above the error bars. Peak latencies remained consistent with those observed in **Figure 5B**, indicating that frame averaging does not shift temporal encoding dynamics.

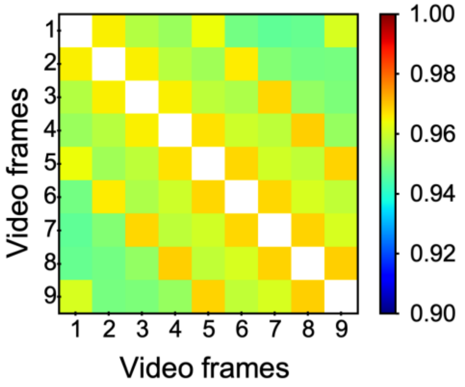

Supplementary Figure 3. Pairwise similarity across video frames. To quantify the similarity among the 9 frames within each video, we computed the Structural Similarity Index Metric (SSIM; Wang et al., 2004) for all frame pairs, then averaged across all videos in the stimulus set to obtain a representational similarity matrix. The results show consistently high similarity across frames, suggesting that successive frames capture redundant visual information.

Supplementary Note 4. Task effects on encoding analysis

In the experiment, participants were instructed to detect whether one of three target characters was playing the guitar. While the target character varied across runs, the target action, playing the guitar, remained constant. To assess potential task-related effects on encoding, we repeated the main analysis after excluding all trials in which a person was playing the guitar. Results are shown in **Supplementary Figure 4**, separately for images and videos.

Removing guitar trials produced qualitative and quantitative changes in skeleton position and action identity for both images and videos, the two features directly linked to the target action. Specifically, the second peak around 500-600 ms present in the original data disappeared for images, and the encoding curves for both features became narrower for images and videos. This shift is also reflected in peak latency estimates: for action identity, latency decreased from 540 ms to 280 ms for images, and for skeleton position (images only), confidence intervals shifted similarly.

These results suggest that two of the seven features, skeleton position and action identity, are sensitive to task-related modulation in the image condition.

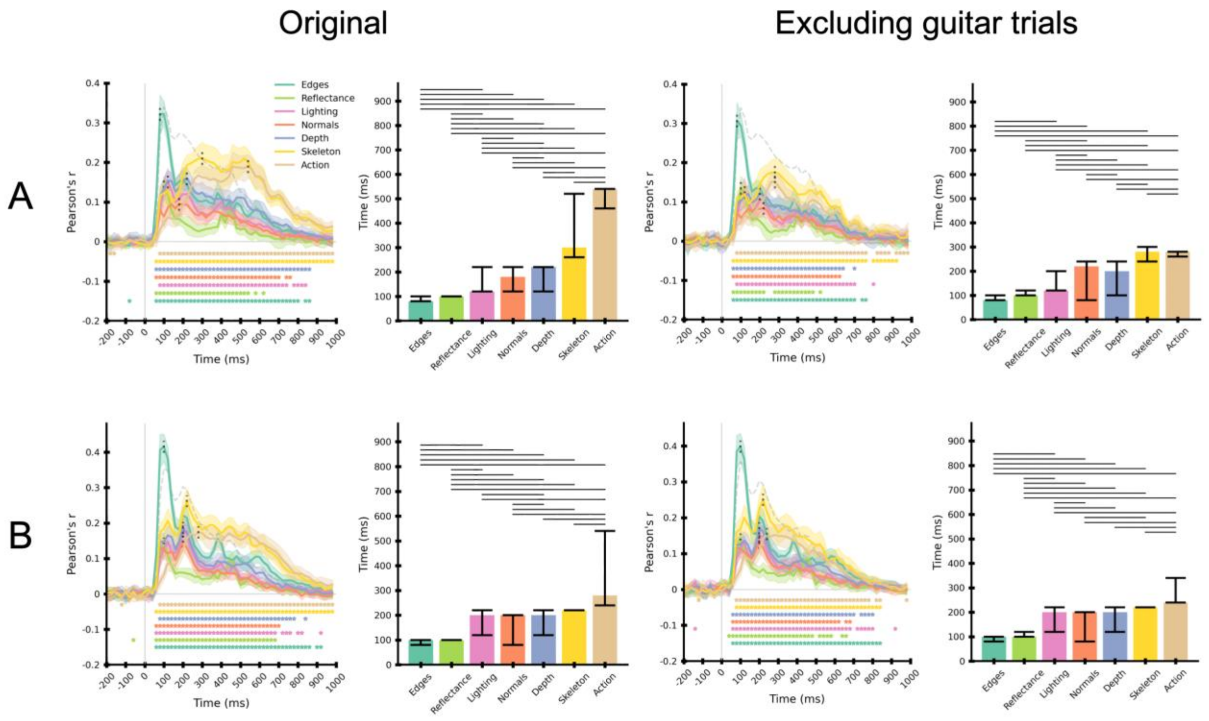

Supplementary Figure 4. Replication of EEG encoding analysis excluding trials with guitar-playing characters. Encoding time courses for EEG responses collected during the viewing of (**A**) images (15 subjects) and (**B**) videos (20 subjects). Results of the main analysis are shown on the left, and results excluding all guitar-playing trials on the right. Left panel: the time course of one low-, five mid-, and one high-level feature representations. We indicated the significant time points with stars (two-tailed, $p \leq 0.05$, FDR-corrected), the 95% confidence intervals with shaded areas around the curves, the stimulus onset with a vertical gray line, the chance level with a horizontal gray line and the noise ceiling with a shaded gray area. Right panel: peak latencies of the features. We indicated the 95% confidence intervals with vertical error bars, the significance ($p \leq 0.05$) between feature peak latencies with horizontal bars, and the significant peak latency differences with stars above the error bars.

Supplementary Note 5. Differences in encoding results for images and videos

Aside from later peak latencies for skeleton position and action identity, we observed no significant differences in the EEG encoding time courses for images versus videos (**Figure 5C**). To investigate this further, we examined whether the similarity in encoding results could be attributed to the high correlation between image and video features. Pearson's correlations were computed between image ground-truth annotations (frame 10) and video annotations (frames 1-9 averaged) across all stimuli.

As shown in **Supplementary Figure 5**, correlations between image and video features were consistently high ($r>0.8$) for most feature types, indicating substantial overlap in the annotations. The exception was low-level edge features, which showed a comparatively low correlation ($r \approx 0.15$). This likely reflects the sensitivity of the Canny

algorithm (Canny, 1986) to small spatial variations, combined with the effect of averaging across frames, which tends to blur sharp edges and reduce their consistency over time.

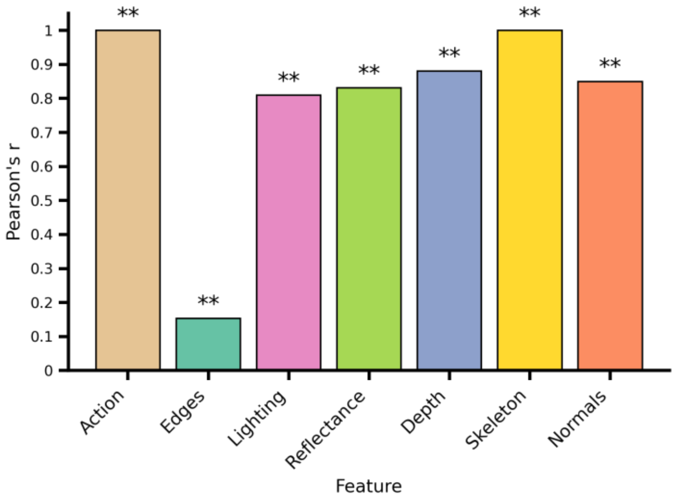

Supplementary Figure 5. Pearson's correlation between image and video ground-truth annotations. Correlations across all stimuli in the training, validation, and test sets indicate a strong similarity ($r>0.8$) for all features except the low-level edge feature. Significance stars indicate $p \leq 0.05$ (*), $p \leq 0.01$ (**), and $p \leq 0.001$ (***). P-values were computed using permutation tests with 1,000 permutations as implemented in scipy.stats.

To test whether this similarity in ground-truth annotations contributed to the similarity in EEG predictions, we used image annotations to predict EEG responses to videos. As shown in **Supplementary Figure 6**, this did not meaningfully change the encoding curves or peak latencies. We did observe a small decrease in encoding accuracy at early time points for the low-level edge feature when using image annotations to predict video responses. Overall, these results suggest that the lack of differences between image and video encoding may be partly explained by the similarity of the ground-truth annotations, which likely arises from both averaging features across frames and the relatively short videos (300 ms), in which many mid-level features show limited variation.

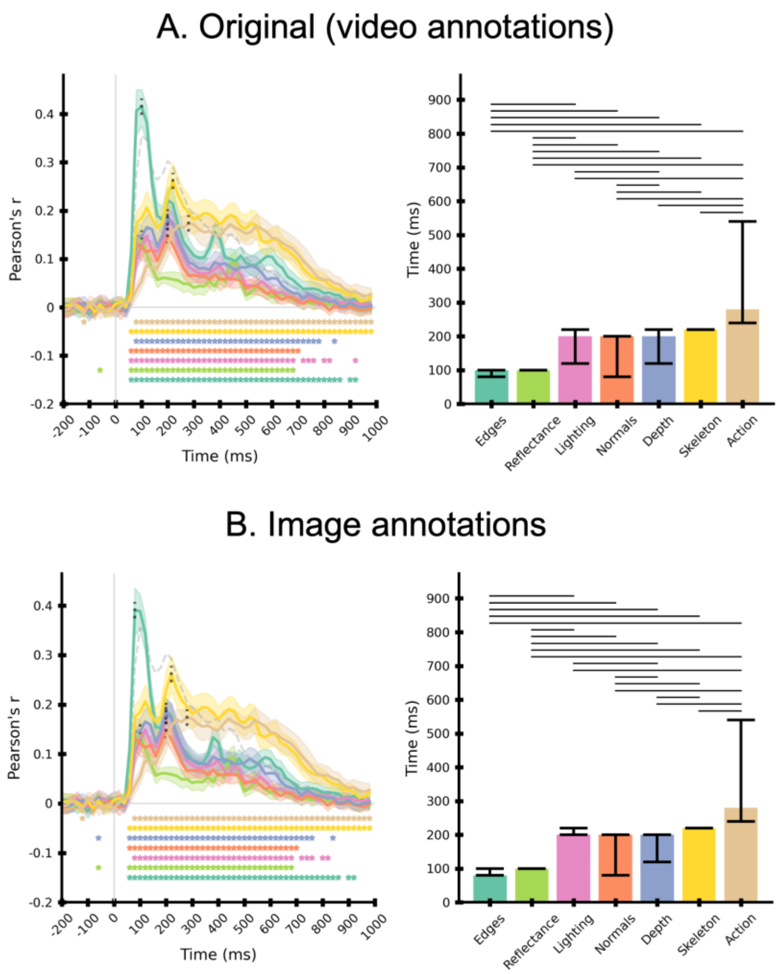

Supplementary Figure 6. Replication of video EEG encoding analysis using image annotations. Encoding time courses for the EEG responses predicted from ground-truth annotations extracted from (**A**) video frames (main analysis) and (**B**) static images. Note that this static image was not extracted from the video itself, but was the frame immediately following the video. Left panel: the time course of one low-, five mid-, and one high-level feature representations. We indicated the significant time points with stars (two-tailed, p≤0.05, FDR-corrected), the 95% confidence intervals with shaded areas around the curves, the stimulus onset with a vertical gray line, the chance level with a horizontal gray line and the noise ceiling with a shaded gray area. Right panel: peak latencies of the features. We indicated the 95% confidence intervals with vertical error bars, the significance (p≤0.05) between feature peak latencies with horizontal bars, and the significant peak latency differences with stars above the error bars. Overall, we observed results highly similar to those in Figure 5b.

Supplementary Note 6. Effects of training objective on CNN encoding results

In the main manuscript, we found that the action feature most strongly predicted unit activations in mid-level layers of a 2D ResNet-18 (He et al., 2016) and in late layers of a 3D ResNet-18 (Tran et al., 2018). Since the 2D model was trained on scene classification (using images) and the 3D model on action classification (using videos), we speculated that although the models differed in input modality, architecture and training objective, this difference is mostly driven by the training objective. To determine if that is the case, we performed a control analysis by comparing two models of the same architecture (2D ResNet-18) that were fed inputs of the same modality (images) but were trained on different tasks (action classification vs scene classification). Since we already had the results for the 2D ResNet-18 model trained on scene classification, we only needed to train a 2D ResNet-18 model on an action classification task using images, i.e., center frames from the Kinetics-400 dataset (Kay et al., 2017).

To obtain the center frames from the Kinetics-400 dataset, each video was decoded using the Decord library, and the center frame (i.e., the middle temporal index) was extracted as a static RGB image. Frames were converted to PIL format and preprocessed using standard torchvision transformations: resizing the shorter side to 256 pixels, center-cropping to 224 x 224 pixels, and normalizing using ImageNet mean and standard deviation. The final fully connected layer of the ResNet-18 was replaced to output logits for 400 action classes.

Training was conducted using the Adam optimizer (Kingma & Ba, 2017) with a learning rate of 3e-4 and a batch size of 128. Cross-entropy loss was used as the training objective. The model was trained for 70 epochs, and performance was monitored via validation loss and accuracy via TensorBoard. The best-performing checkpoint based on validation loss was saved for subsequent analysis. All implementation and training were performed using PyTorch and PyTorch Lightning. Importantly, our goal was not to maximize task performance; action recognition from single static frames is known to be challenging, but rather to obtain learned representations suitable for downstream encoding analysis. The training and validation accuracies and losses are shown in **Supplementary Figure 7**.

After training, we repeated the CNN encoding analysis described in Section 2.6 using this action-trained 2D ResNet-18. The results are shown in **Supplementary Figure 8**. While overall encoding accuracy was comparable to that observed with 2D ResNet-18 trained on scene classification (**Figure 6A**), the peak encoding layers for several features shifted: edges peaked later (layer 4.0 vs. 3.0), depth shifted from layer 3.1 to 4.0, and action from 3.1 to 4.1, whereas skeleton peaked earlier (layer 4.0 vs. 4.1). The

peak latencies of reflectance, lighting, and normals remained unchanged. These results suggest that the training objective plays a key role in shaping the processing hierarchy within deep neural networks.

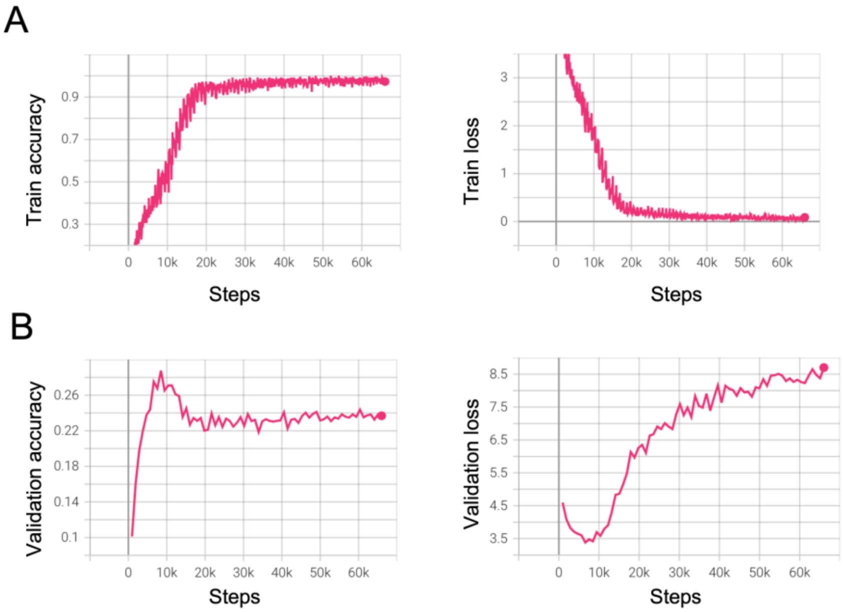

Supplementary Figure 7. Performance on training and validation set of the 2D ResNet-18 used in the CNN encoding analysis. Training and validation performance of the 2D ResNet-18 trained from scratch on action classification using the Kinetics-400 dataset. (**A**) Training accuracy (left) and training loss (right) plotted over training steps. Accuracy increased steadily and plateaued above 90%, while the loss decreased to near zero, indicating effective learning on the training set. (**B**) Validation accuracy (left) and validation loss (right) plotted over training steps. Validation accuracy peaked early (≈0.26) and remained stable, while validation loss decreased initially but subsequently increased, indicating overfitting. The final model checkpoint was selected based on peak validation loss after around 7 epochs.

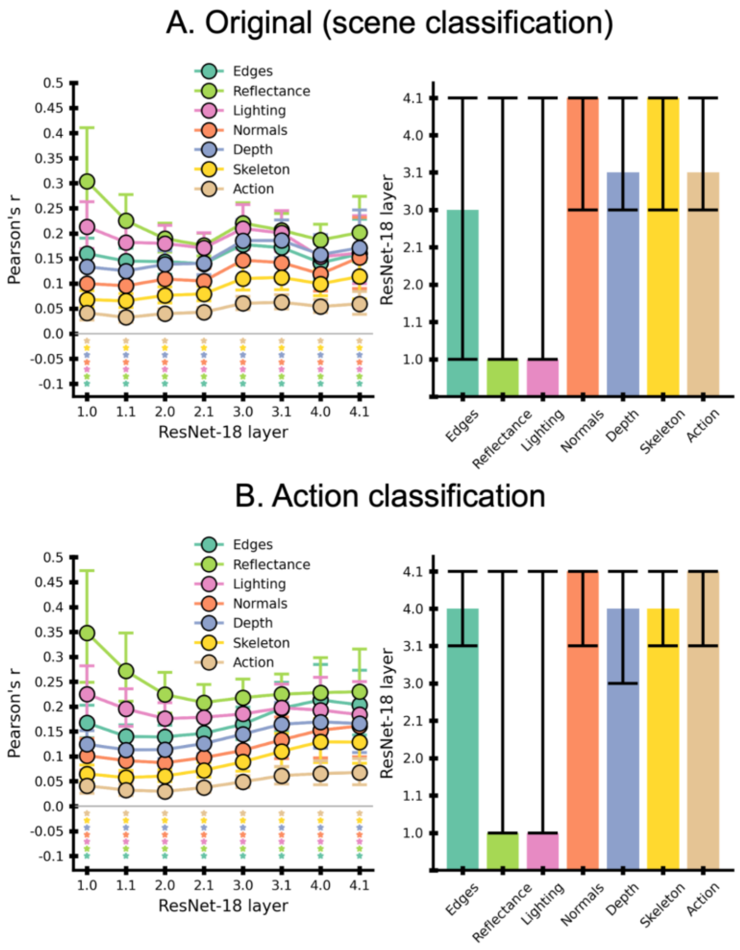

Supplementary Figure 8. Replication of CNN encoding analysis using a 2D ResNet-18 trained on action classification. Encoding results for CNN activations collected during the presentation of images to ResNet-18 trained on (**A**) scene classification (main analysis) and (**B**) action classification. Left panel: encoding accuracy for one low-, five mid-, and one high-level feature representations, for eight CNN layers (named 1.0, 1.1, 2.0, 2.1, 3.0, 3.1, 4.0 and 4.1). We indicated the peak layers with dashed lines, the significant layers with stars (two-tailed, p≤0.05, FDR-corrected) and the 95% confidence intervals with shaded areas around the curves. Right panel: peak layers of the encoding accuracies for the seven features. We indicated the 95% confidence intervals with vertical error bars and the significance (p≤0.05) between feature peak layers with horizontal bars. While overall encoding accuracies were comparable to those observed for a 2D ResNet-18 trained on scene classification (main manuscript), the peak encoding layers shifted for several features: edges (4.0 vs. 3.0), depth (4.0 vs. 3.1), and action (4.1 vs. 3.1) peaked later, whereas skeleton peaked earlier (4.0 vs. 4.1). This shift suggests that the training objective substantially influences the hierarchical organization of feature representations in deep neural networks.

Supplementary Table 1. Descriptions of ground-truth feature annotations

Feature	Description
**Lighting**	Lighting is represented as a 520 x 390 matrix with values ranging from 0 to 255. Low values correspond to dark areas (e.g., shadows), and high values correspond to bright areas (e.g., light sources).
**Edges**	Edges are represented as a 520 x 390 matrix with values ranging from 0 to 255. They correspond to locations where brightness changes abruptly (i.e., discontinuities) and were extracted using the Canny edge algorithm (Canny, 1986) in OpenCV.
**Skeleton position**	Skeleton positions are represented as a 14 x 2 matrix containing the 2D coordinates in the image or video frame of 14 body joints: head, neck, right upper arm, right lower arm, right hand, left upper arm, left lower arm, left hand, right thigh, right calf, right foot, left thigh, left calf, and left foot.
**Scene depth**	Depth is represented as a 520 x 390 matrix, with values from 0 to 255. The luminance is proportional to the distance from the camera position, such that darker areas correspond to closer surfaces and brighter areas correspond to more distant surfaces.
**World normals**	World normals are represented as a 520 x 390 x 3 matrix, with values from 0 to 255 and each pixel containing a 3D vector encoding the X, Y, and Z components of the surface normal in 3D space.
**Reflectance**	Reflectance is represented as a 520 x 390 x 3 matrix, with values from 0 to 255. It is a surface property that represents the ratio of reflected to incident light, with each pixel containing the reflectance values for the red, green, and blue channels.
**Action identity**	Action identity is represented as a one-hot vector encoding the action performed by the character. Six actions were included (arm stretching, cheering while sitting, picking up a bottle from the floor, sit-ups, standing up, and playing guitar), with each scene represented by a vector indicating the corresponding action.

Descriptions of the ground-truth visual features extracted from the stimulus images and videos rendered in Unreal Engine (Epic Games, 2019).

Supplementary Note 7. Effects of input resolution on CNN encoding results

One potential explanation for the differences between the 2D and 3D ResNet-18 encoding results is the variation in input resolution: 224 x 224 pixels for the 2D model versus 112 x 112 pixels for the 3D model. To evaluate this, we downscaled the inputs for the 2D ResNet-18 from 224 x 224 to 112 x 112 pixels before extracting unit activations and repeated the encoding analysis described in section 2.6.

As shown in **Supplementary Figure 9**, this rescaling shifted the peak layers for several features: normals (from layer 4.1 to 3.0), depth (from 3.0 to 3.1), skeleton (from 4.1 to 3.0), and action (from 3.1 to 3.0). Together with the findings from **Supplementary Note 6**, these results underscore the importance of systematic comparisons across different network design choices and suggest that peak layers for most features are sensitive to such factors. In contrast, reflectance and lighting consistently predict early CNN layers of 2D ResNet-18 regardless of input resolution, task objective (**Supplementary Note 6**), or hyperparameter optimization (**Supplementary Note 12**).

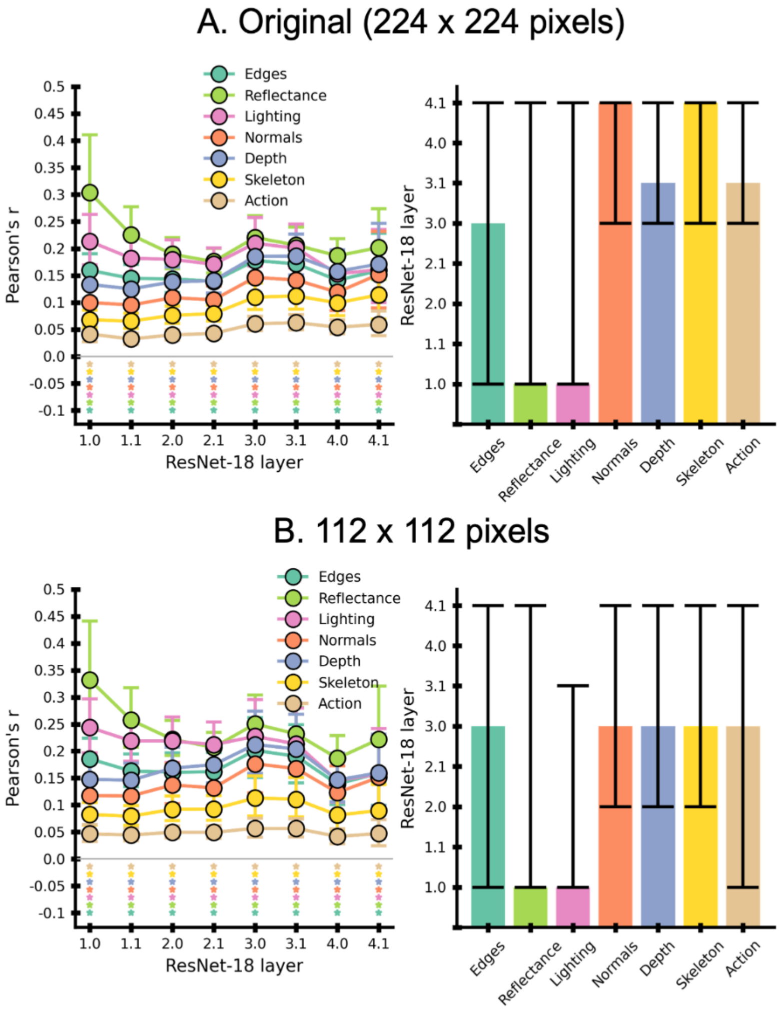

Supplementary Figure 9. Impact of input downscaling on CNN encoding analysis with 2D ResNet-18. Encoding results for CNN activations collected during the presentation of images to ResNet-18 (**A**) with a resolution of 224 x 224 pixels (main analysis) and (**B**) 112 x 112 pixels. Left panel: encoding accuracy for one low-, five mid-, and one high-level feature representations, for eight CNN layers (named 1.0, 1.1, 2.0, 2.1, 3.0, 3.1, 4.0 and 4.1). We indicated the peak layers with dashed lines, the significant layers with stars (two-tailed, p≤0.05, FDR-corrected) and the 95% confidence intervals with shaded areas around the curves. Right panel: peak layers of the encoding accuracies for the seven features. We indicated the 95% confidence intervals with vertical error bars and the significance (p≤0.05) between feature peak layers with horizontal bars. Input downscaling shifted the peak layers of normals (from layer 4.1 to 3.0), depth (from 3.0 to 3.1), skeleton (from 4.1 to 3.0), and action (from 3.1 to 3.0).

Supplementary Note 8. Similarity of ground-truth annotations

Given that many of the candidate mid-level features produced similar encoding time courses and peak latencies, we examined how similar their ground-truth annotations were. This complements the variance partitioning analysis in **Supplementary Note 9**. We assessed representational similarity using two approaches:

- Representational similarity analysis (RSA) across feature pairs
- Centered kernel alignment (CKA) between feature pairs

Representational similarity analysis

We first applied representational similarity analysis (RSA; Kriegeskorte et al., 2008), which does not require ground-truth annotations to share the same representational space or dimensionality. For each feature, we computed a representational dissimilarity matrix (RDM) by calculating 1−Spearman's correlation for every pair of stimuli across the full stimulus set, yielding one RDM of dimensionality #stimuli x #stimuli per feature. We used Spearman's correlation because, unlike Pearson's, it does not assume linear relationships between feature annotations and only requires monotonicity, allowing it to capture possible nonlinearities present in some of the gaming engine's annotations (Kriegeskorte et al., 2008).

Next, we computed 1−Spearman's correlation between each pair of feature RDMs, yielding a second-level RDM with dimensionality #features x #features shown in **Supplementary Figure 10A**. This RDM reflects the similarity between the features' ground-truth annotations.

RSA revealed relatively high similarity ($r \approx 0.2$) between lighting and scene depth, and moderate similarities between skeleton and action, as well as world normals and scene depth. These results align with conceptual similarities, for example, world normals encode depth information, and skeleton position relates to the performed action, and correspond with EEG encoding analysis results (**Figure 5**), where lighting, world normals, and scene depth exhibited similar encoding curves, as did skeleton position and action identity.

Centered kernel alignment

We also applied centered kernel alignment with a linear kernel (CKA; Kornblith et al., 2019) to assess the similarity between the ground-truth annotations. Unlike RSA, which is not fully invariant to orthogonal transformations (e.g., rotations), linear CKA is invariant to linear orthogonal transformations and isotropic scaling. This means linear CKA can detect similarities regardless of rotations or uniform scaling in the feature spaces.

To compute linear CKA, we first centered the Gram matrices (linear kernels) of each annotation pair and then applied the Hilbert-Schmidt Independence Criterion (HSIC) following Kornblith et al. (2019). The resulting similarity scores were organized into a symmetric CKA matrix, visualized as a heatmap in **Supplementary Figure 10B**.

Consistent with the RSA results, CKA revealed moderate to high similarity between scene depth and world normals, and between skeleton position and action identity. Additional relationships were observed between reflectance and lighting, and between skeleton position and scene depth, highlighting further nuanced dependencies. Together, these findings indicate substantial interdependence and redundancy among the chosen visual features.

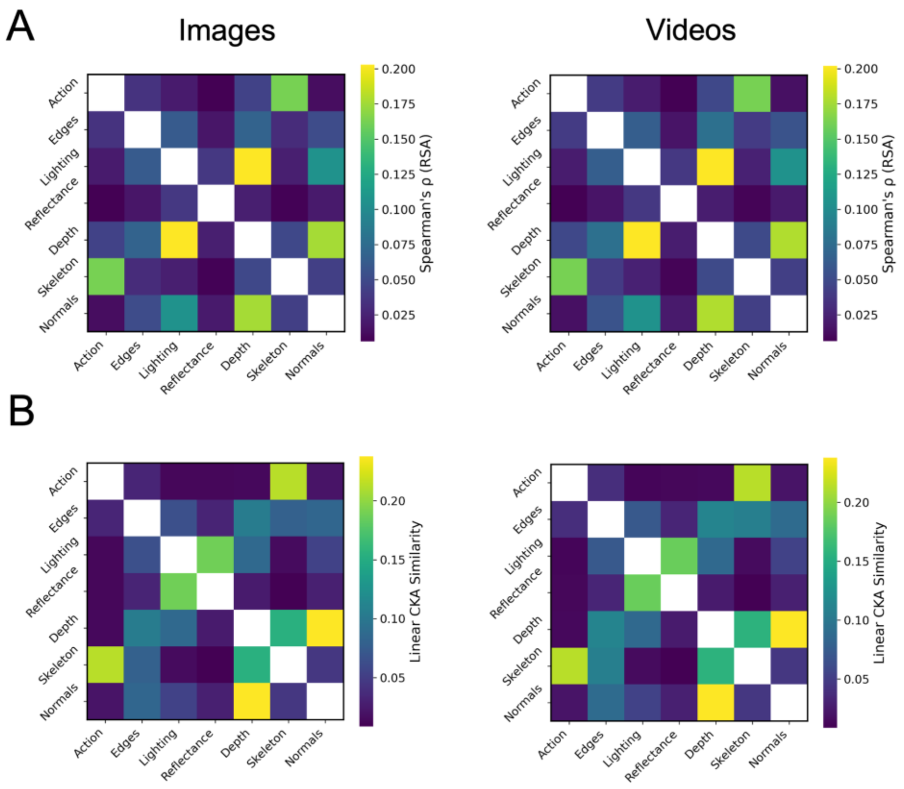

Supplementary Figure 10. Similarity analyses of ground-truth feature annotations. (**A**) Representational similarity analysis (RSA) based on second-level representational dissimilarity matrices (RDMs) revealed similarities between lighting and scene depth, skeleton position and action identity, and world normals and scene depth. (**B**) Linear centered kernel alignment (CKA) confirmed these relationships and uncovered similarities between reflectance and lighting, as well as skeleton and scene depth. Together, these analyses demonstrate substantial interdependence and redundancy among the ground-truth annotations of the candidate mid-level features.

Supplementary Note 9. Variance partitioning analysis

We used variance partitioning to quantify the contributions of each feature, following the procedures of Heer et al. (2017) and Lescroart & Gallant (2019). First, we quantified the unique variance each feature explained separately for image and video data. Second, we assessed how much unique variance each mid-level feature explained beyond the variance already accounted for by the framing features (edges and action).

Unique variance explained by each feature

To determine the unique variance explained by each feature, we defined 8 models:

- A full model including all features as predictors, i.e., edges, lighting, reflectance, scene depth, world normals, skeleton position, and action identity. Concatenating these features resulted in 534 predictors in total.
- Seven leave-one-out models, each excluding one feature at a time (e.g., leaving out edges but including all others).

We then fitted these models as described in the main manuscript (section 2.6). For each model, we computed the explained variance on the test set as the squared Pearson correlation between the predicted and actual EEG responses (Lescroart & Gallant, 2019):

$$\hat{R}_i^2 = \mathrm{r}(Y_i, \hat{Y}_i)^2$$

Thus, here, R^2 reflects the strength of the linear relationship between predicted and observed EEG responses rather than the proportion of variance explained in the classical sense.

In some cases, the full model explained less variance than some individual models, which is theoretically impossible and likely due to overfitting and sampling errors. To correct for this bias, we applied a post-hoc adjustment following Heer et al. (2017). For each time point t and channel c, we collected the uncorrected R^2 values from all individual models and the full model into a vector:

$$\hat{X}_{t,c} = \left[\hat{R}_1^2, \hat{R}_2^2, \ldots, \hat{R}_k^2, \hat{R}_{\mathrm{full}}^2\right]$$

To correct for model estimation bias, we estimated a vector of bias terms $[b_1, b_2, \ldots, b_k, b_{\mathrm{full}}] \in \mathbb{R}^{k+1}$ by solving the following constrained optimization problem:

$$\min_{\mathbf{b}} \sum_{i=1}^{k+1} b_i^2$$

The key constraint was that the full model must explain at least as much variance as each individual model:

$$\hat{R}^2_{\text{full}} + b_{\text{full}} \geq \hat{R}^2_i + b_i \quad \forall i \in \{1, \ldots, k\}$$

These constraints can be expressed as inequality constraints for the optimizer:

$$g_i(\mathbf{b}) = \hat{R}^2_{\text{full}} + b_{\text{full}} - \hat{R}^2_i - b_i \geq 0$$

We used the scipy.optimize.minimize function with the SLSQP method to solve this optimization problem. Once the bias terms were estimated, we computed the unique variance explained by each feature model by adjusting the uncorrected variance values accordingly:

$$\text{Unique Variance}_i = (\hat{R}^2_{\text{full}} + b_{\text{full}} - \hat{R}^2_i - b_i) * 100 \text{ (in \%)}.$$

For illustration, the unique variance explained by a given feature (e.g., edges) corresponds to the adjusted R^2 difference between the full model and the model excluding that feature. Results (**Supplementary Figure 11A,B**) show that most features explained ~1-2.5% unique variance. Lighting explained the most unique variance until ~200-250 ms. Skeleton position consistently accounted for the largest unique variance from ~200-250 ms onwards, while reflectance exhibited a distinct second peak after ~450 ms.

Unique variance explained by each mid-level feature accounting for framing features

To assess the unique variance explained by each mid-level feature beyond the framing features, we defined six models:

- A baseline model including only the framing features (edges and action identity).
- Five models that each add one mid-level feature to the baseline predictors.

We then followed the same procedure as described above to compute the explained variance R^2 for each model. The full model in this case is the baseline model consisting of only the framing features. While the optimization problem remains the same, we adapted the constraint such that each individual model must explain at least as much variance as the baseline model:

$$\hat{R}_i^2 + b_i \geq \hat{R}_{\text{baseline}}^2 + b_{\text{baseline}} \quad \forall i \in \{1, \ldots, k\}$$

This constraint can be expressed as inequality constraints for the optimizer:

$$g_i(\mathbf{b}) = \hat{R}_i^2 + b_i - \hat{R}_{\text{baseline}}^2 - b_{\text{baseline}} \geq 0$$

The unique variance explained by each mid-level feature, while accounting for the framing features, is computed by adjusting the explained variance values accordingly:

$$\text{Unique Variance}_i = (\hat{R}_i^2 + b_i - \hat{R}_{\text{baseline}}^2 - b_{\text{baseline}}) * 100 \text{ (in \%)}.$$

Supplementary Figure 11C shows that skeleton position explains most unique variance across the whole time course in comparison to the other mid-level features. Together, the results indicate that while several mid-level features contribute unique information beyond framing cues, skeleton position provides the most robust and sustained contribution.

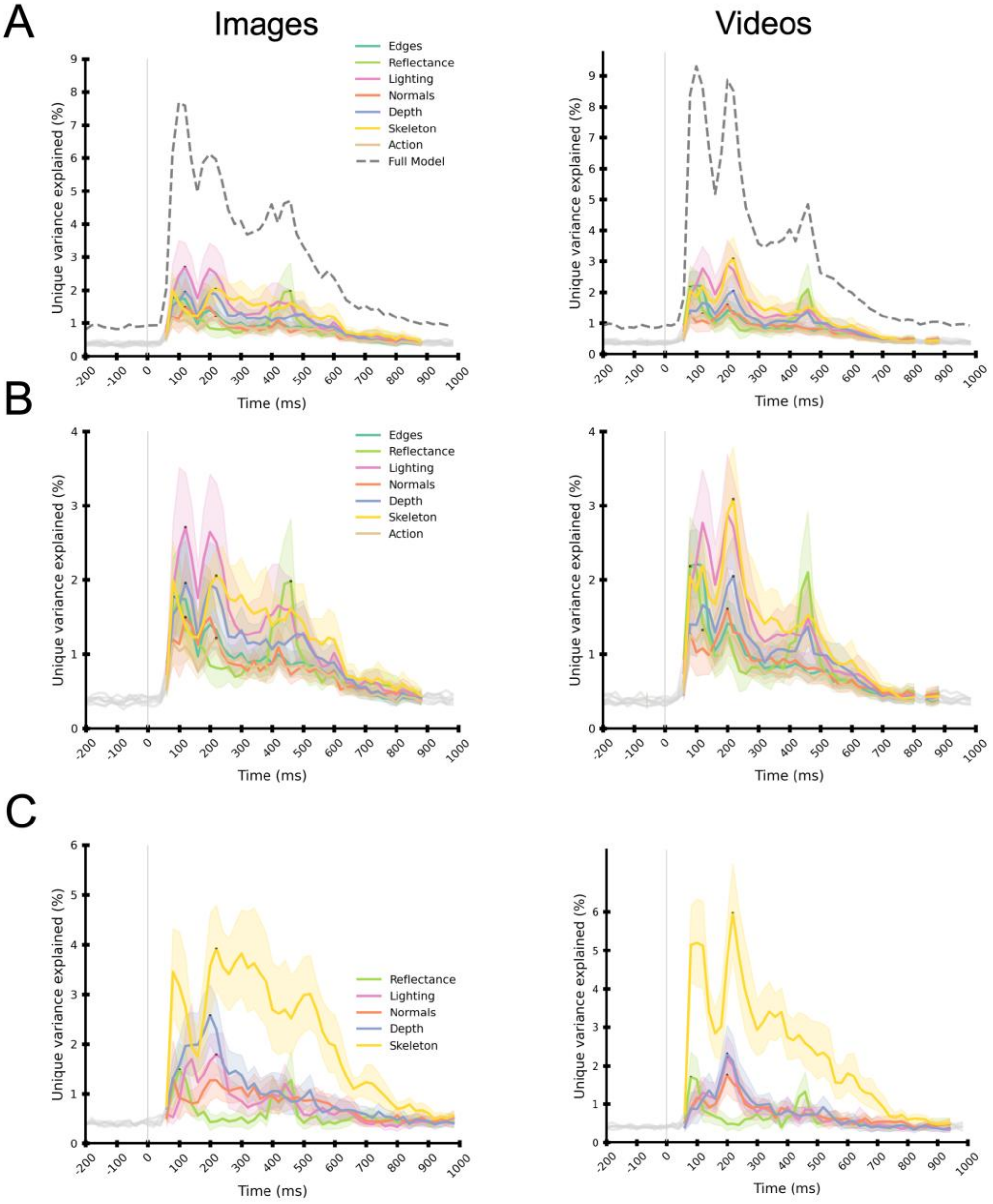

Supplementary Figure 11. Unique variance explained over time. (**A**) Unique variance explained by each feature over time for images (left) and videos (right), computed using variance partitioning analysis (see **Supplementary Note 9** for details). The dashed line indicates the total variance explained by the full model. Gray shading highlights time points where the full model, the model with all features, did not significantly predict the EEG responses (two-tailed, p≤0.05, FDR-corrected). Most features account for 1-2.5% unique variance, with largely overlapping temporal profiles. (**B**) Same data as in (A), with the full model curve (dashed line) omitted for clarity. (**C**) Unique variance explained by mid-level features when controlling for the framing features (edges and action identity), shown for images (left) and videos (right). Skeleton position consistently explains the most unique variance over time. Gray shading indicates non-significant time points based on the baseline model, the model with only the framing feature (two-tailed, p≤0.05, FDR-corrected). Critically, small non-zero baseline values in A-C are expected due to the bias-correction procedure and the computation of R^2, and are included only as a visual reference for the magnitude of the post-stimulus effect. Interpretation should be restricted to coloured time points.

Supplementary Table 2. Statistical details for the encoding analysis on EEG data

	Significant time points (ms)	Peak latency [95% CI] (ms)
**<u>Images</u>**		
Edges	-80, 60:800, 840:860	80 [80, 100]
Reflectance	60:540, 580, 620	100 [100, 100]
Lighting	80:740, 780:840	120 [120, 220]
World normals	60:700, 740:760	180 [120, 220]
Scene depth	-360, 60:860	220 [120, 220]
Skeleton position	60:980	300 [260, 520]
Action identity	-180:-160, 80:980	540 [460, 540]
**<u>Videos</u>**		
Edges	60:880, 900:920	100 [80, 100]
Reflectance	-260, -60, 60:680	100 [100, 100]
Lighting	60:680, 720:760, 800:820, 920	200 [120, 220]
World normals	60:700	200 [80, 200]
Scene depth	80:780, 840	200 [120, 220]
Skeleton position	60:980	220 [220, 220]
Action identity	-120, 80:980	280 [240, 540]
**<u>Difference</u>**		
Edges	100:120, 200:220	20 [0, 20]
Reflectance	-	0 [0, 20]
Lighting	-	80 [0, 100]
World normals	-	20 [0, 140]
Scene depth	-	20 [0, 100]
Skeleton position	-	80 [40, 300]

Action identity	-	260 [0, 300]

Significant time points, peak latencies with their 95% confidence intervals (10,000 bootstrap iterations over participants) and peak values for the encoding analysis on image and video EEG data.

Supplementary Table 3. Number of principal components used to capture 90% or more of the variance in the CNN encoding analysis

	**Number of components**
**Image CNN**	
Layer 1.0	779
Layer 1.1	840
Layer 2.0	848
Layer 2.1	856
Layer 3.0	768
Layer 3.1	788
Layer 4.0	795
Layer 4.1	575
**Video CNN**	
Layer 1.0	849
Layer 1.1	862
Layer 2.0	844
Layer 2.1	847
Layer 3.0	776
Layer 3.1	785
Layer 4.0	771
Layer 4.1	628

Supplementary Note 10. Lower and upper noise ceilings for the encoding analysis

In Figure 5, we only depicted the lower noise ceiling, to ensure that the feature-specific time courses are well visible. Here, we include the upper noise ceiling (calculated as described in the Methods section), for the encoding time courses from the image experiment (**Supplementary Figure 12A**) and the video experiment (**Supplementary Figure 12B**).

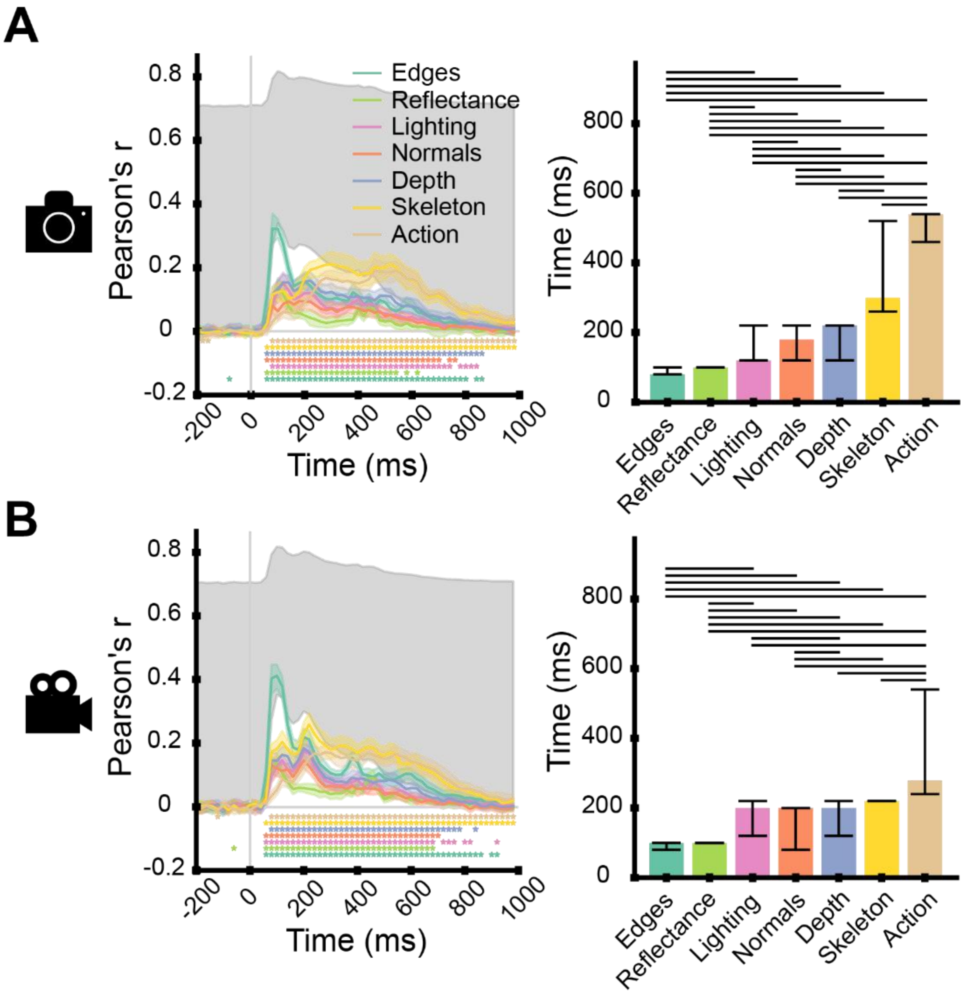

Supplementary Figure 12. EEG encoding results with upper and lower noise ceilings. Encoding time courses for EEG responses collected during the viewing of (**A**) images (15 subjects) and (**B**) videos (20 subjects). Left panel: the time course of one low-, five mid-, and one high-level feature representations. We indicated the significant time points with stars (two-tailed, p≤0.05, FDR-corrected), the 95% confidence intervals with shaded areas around the curves, the stimulus onset with a vertical gray line, the chance level with a horizontal gray line and the noise ceiling with a shaded gray area between the lower and the upper noise ceilings. Right panel: peak latencies of the feature-specific encoding curves. We indicated the 95% confidence intervals with vertical error bars and the significance (p≤0.05) between feature peak latencies with horizontal bars.

References

Canny, J. (1986). A Computational Approach to Edge Detection. *IEEE Transactions on Pattern Analysis and Machine Intelligence*, *PAMI-8*(6), 679–698. IEEE Transactions on Pattern Analysis and Machine Intelligence. https://doi.org/10.1109/TPAMI.1986.4767851

Cichy, R. M., Pantazis, D., & Oliva, A. (2014). Resolving human object recognition in space and time. *Nature Neuroscience*, *17*(3), Article 3. https://doi.org/10.1038/nn.3635

Epic Games. (2019). *Unreal Engine* (Version 4.22.1) [Computer software]. https://www.unrealengine.com

He, K., Zhang, X., Ren, S., & Sun, J. (2016). Deep Residual Learning for Image Recognition. *2016 IEEE Conference on Computer Vision and Pattern Recognition (CVPR)*, 770–778. https://doi.org/10.1109/CVPR.2016.90

Heer, W. A. de, Huth, A. G., Griffiths, T. L., Gallant, J. L., & Theunissen, F. E. (2017). The Hierarchical Cortical Organization of Human Speech Processing. *Journal of Neuroscience*, *37*(27), 6539–6557. https://doi.org/10.1523/JNEUROSCI.3267-16.2017

Isik, L., Tacchetti, A., & Poggio, T. (2018). A fast, invariant representation for human action in the visual system. *Journal of Neurophysiology*, *119*(2), 631–640. https://doi.org/10.1152/jn.00642.2017

Johansson, G. (1973). Visual perception of biological motion and a model for its analysis. *Perception & Psychophysics*, *14*(2), 201–211. https://doi.org/10.3758/BF03212378

Kay, W., Carreira, J., Simonyan, K., Zhang, B., Hillier, C., Vijayanarasimhan, S., Viola, F., Green, T., Back, T., Natsev, P., Suleyman, M., & Zisserman, A. (2017). *The Kinetics Human Action Video Dataset* (arXiv:1705.06950). arXiv. https://doi.org/10.48550/arXiv.1705.06950

King, J.-R., & Dehaene, S. (2014). Characterizing the dynamics of mental representations: The temporal generalization method. *Trends in Cognitive Sciences*, *18*(4), 203–210. https://doi.org/10.1016/j.tics.2014.01.002

Kingma, D. P., & Ba, J. (2017). *Adam: A Method for Stochastic Optimization* (arXiv:1412.6980). arXiv. https://doi.org/10.48550/arXiv.1412.6980

Kornblith, S., Norouzi, M., Lee, H., & Hinton, G. (2019). *Similarity of Neural Network Representations Revisited* (arXiv:1905.00414). arXiv. https://doi.org/10.48550/arXiv.1905.00414

Kriegeskorte, N., Mur, M., & Bandettini, P. A. (2008). Representational similarity analysis—Connecting the branches of systems neuroscience. *Frontiers in Systems Neuroscience*, *2*. https://doi.org/10.3389/neuro.06.004.2008

Lescroart, M. D., & Gallant, J. L. (2019). Human Scene-Selective Areas Represent 3D Configurations of Surfaces. *Neuron*, *101*(1), 178-192.e7. https://doi.org/10.1016/j.neuron.2018.11.004

Tran, D., Wang, H., Torresani, L., Ray, J., LeCun, Y., & Paluri, M. (2018). *A Closer Look at Spatiotemporal Convolutions for Action Recognition*. 6450–6459. https://openaccess.thecvf.com/content_cvpr_2018/html/Tran_A_Closer_Look_CVPR_2018_paper.html

Wang, Z., Bovik, A. C., Sheikh, H. R., & Simoncelli, E. P. (2004). Image quality assessment: From error visibility to structural similarity. *IEEE Transactions on Image Processing*, *13*(4), 600–612. IEEE Transactions on Image Processing. https://doi.org/10.1109/TIP.2003.819861
